# On the use of atomistic simulations to aid bulk metallic glasses structural elucidation with solid-state NMR

**DOI:** 10.1038/s41598-017-08919-6

**Published:** 2017-08-24

**Authors:** Ary R. Ferreira, José P. Rino

**Affiliations:** Department of Physics, Universidade Federal de São Carlos (UFSCar), São Carlos–SP, 13565–905 Brazil

## Abstract

Solid-state nuclear magnetic resonance (ssNMR) experimental ^27^Al metallic shifts reported in the literature for bulk metallic glasses (BMGs) were revisited in the light of state-of-the-art atomistic simulations. In a consistent way, the Gauge-Including Projector Augmented-Wave (GIPAW) method was applied in conjunction with classical molecular dynamics (CMD). A series of Zr-Cu-Al alloys with low Al concentrations were selected as case study systems, for which realistic CMD derived structural models were used for a short- and medium-range order mining. That initial procedure allowed the detection of trends describing changes on the microstructure of the material upon Al alloying, which in turn were used to guide GIPAW calculations with a set of abstract systems in the context of ssNMR. With essential precision and accuracy, the *ab initio* simulations also yielded valuable trends from the electronic structure point of view, which enabled an overview of the bonding nature of Al-centered clusters as well as its influence on the experimental ssNMR outcomes. The approach described in this work might promote the use of ssNMR spectroscopy in research on glassy metals. Moreover, the results presented demonstrate the possibility to expand the applications of this technique, with deeper insight into nuclear interactions and less speculative assignments.

## Introduction

The quest for a better understanding of composition-structure-property relationships is one of the main focus in materials science research. Regarding structural characterization, solid-state nuclear magnetic resonance (ssNMR) spectroscopy is a powerful technique that has been employed with a wide range of materials. Among them, those which can be obtained as amorphous solids with well-defined glass transition temperatures (*T*_*g*_) are particularly challenging. This is because the vitrification process yields a product which structure is often described as a snapshot of the precursor liquid^1^, but naturally without motional averaging of the anisotropic nuclear interactions. Thus, due to chemical and topological disorder, the inherent powder line shape observed in the spectra of glasses is far from the high-resolution counterparts taken for crystalline samples. Therefore, even with great breakthroughs in the field of ssNMR, it is still difficult to report unambiguous peak assignments without the aid of computational simulations. In this context, the Gauge-Including Projector Augmented-Wave (GIPAW) method^2, 3^ was introduced in the last decade, and nowadays it is possible to state that its use for the computation of ssNMR spectral parameters has become routine for non-metallic materials, including glasses^4, 5^. Moreover, since the GIPAW method is based on the Density Functional Theory (DFT)^6, 7^, the computed ground state charge density can guide further chemical bonding analysis.

Metallic amorphous solids are not exactly a new class of materials, as they were reported for the first time in the late 1950s^8^. However, since then, effective research and development into their processing and practical applications have been delayed for almost three decades. Among other key aspects of the synthesis process, the high cooling rates (CRs) needed to produce those alloys, in such a way to stop the atomic mobility before crystallization, have greatly contributed to that backwardness. Notwithstanding, presently bulk metallic glasses (BMGs) with critical diameters in excess of 1 cm are commonly obtained in a wide variety of compositions. In addition to the most pronounced mechanical features like high strength and good toughness^1, 9, 10^, which have established BMGs as promising structural materials, there is also the prospect of their further use as functional materials. Recent works have reported investigations on the magnetic properties of certain glassy alloys^11^ as well as some catalytic activity in electrochemical processes in the scope of energy storage and conversion^12^.

Another important point regarding research and design of BMGs is the limited set of experiments that can be resorted for characterization down to the atomic-scale. This is essential to uncover the structural features that contributes to glass formation ability and properties. Most common techniques are fluctuation and transmission electron microscopies, X-ray and neutron diffraction, and also X-ray absorption fine structure, all usually aided by molecular dynamics (MD) simulations or reverse Monte Carlo modeling^9^. Although the traditional atomic packing perspective of the structure of BMGs has been able to describe their mechanical properties very well, it is intuitive and well accepted that bonding nature also matters. It has been shown that this complementary vision from the electronic structure point of view can be achieved with ssNMR and electron energy loss spectroscopies (EELS)^13, 14^.

The use of ^27^Al ssNMR for the characterization of intermetallic compounds is relatively well established^15^. Recently, it also has been used in a number of studies aimed to establish correlations between peak positions in the measured spectra and mechanical properties of BMGs^13, 14, 16, 17^, or even to understand liquid-liquid transitions in glass forming melts^18^. Nevertheless, as usual and sometimes inevitable in ssNMR studies of glasses, a certain speculative element exists in establishing such relationships in terms of the local/semi-local atomic arrangement and electron density around target sites. In the case of non-metallic glasses, the most prominent shifts of the resonance frequencies are due to the diamagnetic shielding and paramagnetic de-shielding effects, the so-called chemical shifts (CS). However, for metallic systems there is a complicating factor due to the paramagnetism of the conduction electrons, and additional shifts arise from the hyperfine interaction between their spin-polarized magnetic moments and the nuclear spins. These are also known as Knight shifts (KS) and, for some systems, find arguments to separate them from the competing CS without the aid of computational simulations may not be a simple task^19^.

The aim of this work is to introduce the potential of using the GIPAW method associated to classical MD (CMD) simulations to support the interpretation of ssNMR spectra of BMGs. The CMD simulations are primarily used for short- and medium-range order (SRO and MRO) mining in a set of case study systems, for which experimental ^27^Al ssNMR data have recently been made available in the literature^16^. From the CMD derived structural models, conventional statistics allowed us to discover the main features of the SRO and MRO around the Al nuclei which are most likely to justify the experimental metallic shifts (CS plus KS). Then, following a recent use of the GIPAW method to quantify the CS and KS isotropic values in intermetallics compounds from first principles^20^, we apply it to a set of abstract systems (ASs) in the scope of ssNMR. From the results obtained, it was possible to revisit the correlations between metallic shifts and local/semi-local structure around Al sites proposed in the literature.

## Results and Discussion

### Computational approach and case study systems

We start from the premise that CMD is the most appropriate method to create realistic structural models for BMGs. It is expected that, in most of the cases, the description of SRO and MRO in this class of materials requires MD derived large-scale structures with some thousands of atoms. Furthermore, the relatively low quenching rates reported in the literature^21^ suggests the need of large time scales as well. It is also worth mentioning that, taking into account the rather limited time steps in the time integration schemes, due to simulations at high temperatures, it is possible to rule out the use of *ab initio* MD.

The computations of the ssNMR spectral parameters for metals with the GIPAW method, in contrast, demands a quantum mechanical approach that is computationally expensive and quite restricted in the size and asymmetry of the systems at issue. The parameters concerned are associated to the effect of internal anisotropic interactions, which affect the nuclear spins, on the line-shapes and shifts of the resonance frequencies observed in the measured spectra. For intermetallics, the parameters that are extracted by spectral curve fitting, are the metallic shifts (CS plus KS) and the ones related to the quadrupolar interaction with the electric field gradient at the nuclear positions. The quantitative separation of CS and KS by GIPAW calculations is inherent to the method and each contribution is represented by two second-rank tensors named orbital (*σ*_*o*_) and spin (*σ*_*s*_). Fortunately, they can be computed independently of each other and more details regarding theory and technicalities on their simulations can be found in ref. 20.

A second premise stated by us is about the short-ranged structural information obtained from ssNMR experiments. We use it to overcome the impossibility of calculating *σ*_*o*_ and *σ*_*s*_ directly from the CMD derived large-scale structural models of a BMG, by proposing a set of ASs in the scope of ssNMR, from which these two properties can be extrapolated in a consistent way. These ASs may be crystal phases that compete with the BMG under study, or artificial supercells derived from their structures, containing a relatively smaller number of atoms. The extrapolations must be grounded on observed trends or empirical correlations between ssNMR spectral parameters and indexes associated to local/semi-local structure around target nuclei. In fact, this is a strategy that has been used with the GIPAW method to interpret the spectra of non-metallic glasses^22^.

We choose the ternary alloy Zr-Cu-Al (ZCA) as a case study system and focused on the experimental ^27^Al ssNMR isotropic metallic shifts reported by Xi *et al*.^16^ for a set of BMGs with nominal compositions Zr_(50−0.5*x*)_Cu_(50−0.5*x*)_Al_*x*_, with *x* = 2, 4, 6, 8, and 10. From this point, we highlight to the reader that we will label this specific set of BMGs along the text as ZCA-*x*. Additionally, for the sake of comparison, we considered two other alloys of that same series with *x* = 12 and 14 and also the system Zr_47_Cu_46_Al_7_. Given the fixed Zr/Cu ratio, these BMGs provide a systematic overview of the effect of alloying on the electronic and atomic structures around target Al sites, i.e., how the alloying element Al induces changes into the microstructure of the material.

### Short- and medium-range order mining for case study systems

Before starting the CMD simulations for the BMGs ZCA-*x*, one of the tests made by us was to study the effect of different cooling rates (CR) on the structure of the BMG Zr_47_Cu_46_Al_7_. That system was chosen since it was assumed as a case study by Cheng *et al*.^23, 24^ when developing the interatomic potential adopted by us in the present work, which is based on the embedded atom model (EAM) and was validated extensively by the authors. The reader is referred to the supplementary information for detailed results from these tests, and to the Methods section for technical details on our CMD simulations. Based on the assumptions stated in the supplementary information, we justify the use of a minimal and feasible CR of 8.5 × 10^9^ K/s, equivalent to quench the supercooled liquid from 2000 K to 300 K for 200 ns in the time evolution of the MD simulation (Δt).

**Figure 1:**
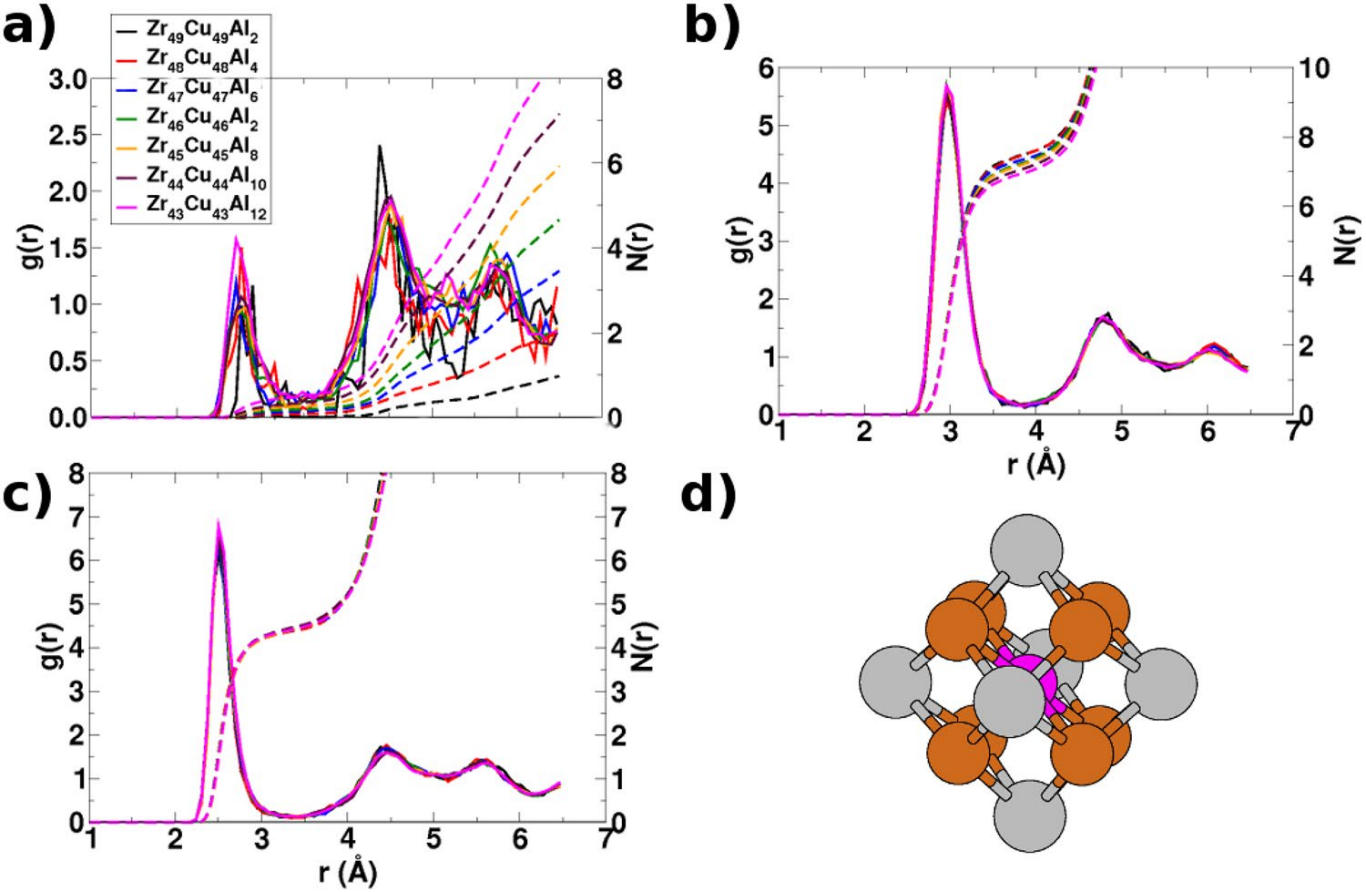
Partial radial distribution functions for the (**a**) Al-Al, (**b**) Al-Zr, and (**c**) Al-Cu pairs computed for the BMGs ZCA-*x*, all prepared with a CR of 8.5 × 10^9^ K/s. The respective integrals are also shown as dashed lines with the same colors. In (**d**) the rhombic dodecahedron coordination polyhedron around the central Al site in the structure of the ZrCu_2_Al *Heusler* phase. In that and in all figures that follows in this document, the colors of Zr, Cu, and Al atoms are gray, brown, and pink, respectively.

We show in Fig. 1 the partial radial distribution functions (PRDFs) for the Al-Al, Al-Zr, and Al-Cu pairs computed for BMGs ZCA-*x*. From which, on a first glance, it is reasonable to state that the SRO around Al sites is virtually the same for all, whereas the MRO changes significantly upon Al alloying. Moreover, the nearest-neighbor distances indicated by the positions of the first maximums in Fig. 1(b,c), are quite compatible with the respective distances d(Al-Zr) = 3.11 Å and d(Al-Cu) = 2.69 Å found in the crystalline phase ZrCu_2_Al. This is one of the main intermetallics competing with glass formation on cooling of these alloys, and it crystallizes in space group $Fm\bar{3}m$, with the Zr, Cu, and Al atoms occupying the 4*a*, 8*c*, and 4*b* Wyckoff positions, respectively. It is possible to analyze the structure of that crystal in terms of a single type of Al-CC, with a rhombic dodecahedron (RD) coordination polyhedron (CP) around the Al center, as shown in Fig. 1(d), with the FCS composed by two concentric CPs: a cube formed by 8 Cu atoms and an octahedron formed by 6 Zr atoms. The integrals of the first peaks of *g*_*AlCu*_(**r**) PRDFs suggest a smaller number of Cu atoms between 4 and 5 in the FCS for all nominal compositions. In contrast, the same analysis for *g*_*AlZr*_(**r**) indicates an overestimation of the number of Zr atoms in the FCS that decreases smoothly with Al concentration from about 8 to 7.

Regarding the MRO of the BMGs, it can be inferred from Fig. 1(a) that Al atoms tend to cluster at distances of about 4.50 Å from each other, according to the second peak of the *g*_*AlAl*_(**r**) PRDFs, i.e., they tend to form clusters of up to 5 Al-centered clusters (Al-CCs). This is a rough estimate but quite feasible, since the integrals of the first peaks are really small. What also points to a rather limited amount of Al-CCs with some few Al atoms in the FCS. We must point out that such conception of the MRO in the structures the BMGs ZCA-*x* is also compatible with the ZrCu_2_Al structure, where each Al-CC shares its 12 identical rhombic faces with 12 other equivalent Al-CCs, with distances d(Al-Al) = 4.40 Å.

**Figure 2:**
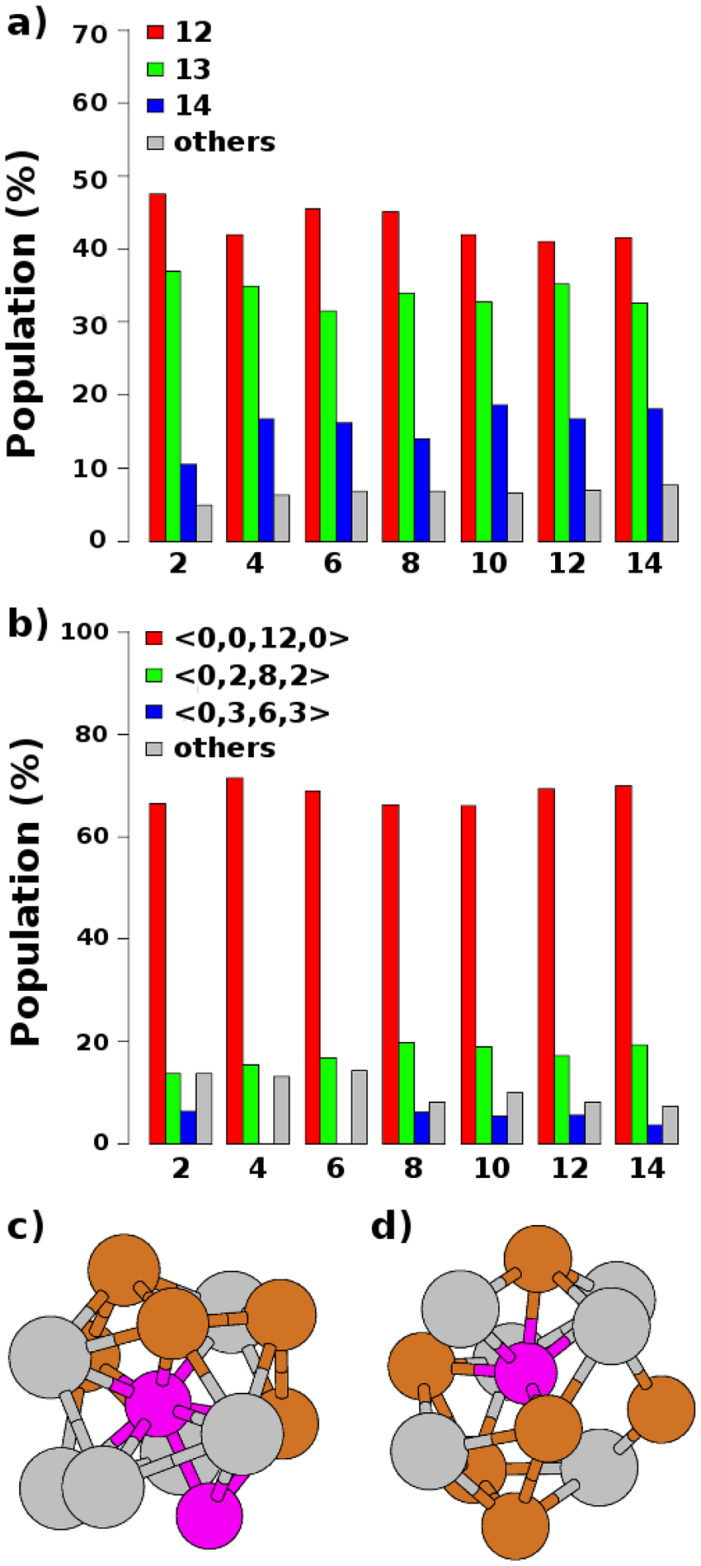
(**a**) Relative populations of Al-centered clusters (Al-CCs) with different coordination numbers computed from Voronoi analyses for each value of *x* in the series of BMGs ZCA-*x*. (**b**) The respective relative populations of Voronoi indexes taking into account only the Al-CCs with coordination number equals to 12. In (**c**,**d**), two examples of Al-CCs with the same Voronoi index 〈0, 0, 12, 0〉.

As done before for the BMG Zr_47_Cu_46_Al_7_, we resort to Voronoi analyses (VA) to gain further insight into the SRO of the BMGs ZCA-*x*. Additionally, we consider the Common Neighbor Analysis (CNA) (see more in ref. 25) as an alternative method, in principle, for the same purpose. We show in Fig. 2(a) that the statistics of coordination numbers (CNs) from VA, supports the assumption that the SRO around the Al sites is virtually the same for all BMGs, including the system Zr_47_Cu_46_Al_7_. What is in agreement with the *g*_*AlAl*_(**r**) PRDFs in Fig. 1(b,c). Even before this scenario, it is worth to look for nuances of the SRO that correlate with the experimental ^27^Al ssNMR isotropic metallic shifts reported by Xi *et al*.^16^ for the BMGs ZCA-*x*. In that work, the authors found an exponential decrease in these shifts, from 320 to 280 ppm, with increasing Al concentration between *x* = 2 and *x* = 10. The VA offers a more precise description of the SRO around each Al-CC, through an index that is based on the frequency of faces with the same number of edges of the respective Voronoi polyhedra. It is represented as 〈*n*_3_, *n*_4_, *n*_5_, …〉, with *n*_*N*_ the number of faces with *N* edges. For example, the Voronoi index (VI) of a regular icosahedron is 〈0, 0, 12, 0〉 whereas the VI of a rhombic dodecahedron (RD) is 〈0, 6, 0, 8〉 and, naturally, these VIs can vary upon distortions of their respective coordination polyhedra (CPs)^26^. We group only the Al-CCs-12 and show the associated statistics in Fig. 2(b), from which it is noticeable that, as well as the coordination numbers (CNs), the VIs do not bring any evidence of correlation between local structure and experimental metallic shifts. Concerning the Al-CCs-13 and Al-CCs-14, we point that we have found a distribution of VIs substantially more heterogeneous, and just few Al-CCs-14 were classified with the VI 〈0, 6, 0, 8〉 (see supplementary Table S3). Additionally, we show in Fig. 1(c,d) two examples of Al-CCs-12, both with the same VI 〈0, 0, 12, 0〉. From them, it can be seen that the VA has its limitations with regards to its ability to distinguish CPs with the same CN, but different degrees of distortions. We must also mention that we tried to consider a refinement of the VAs by eliminating some tiny surfaces and ultra-short edges by setting their respective thresholds, without significant changes on the statistics shown in Fig. 2(b).

**Figure 3:**
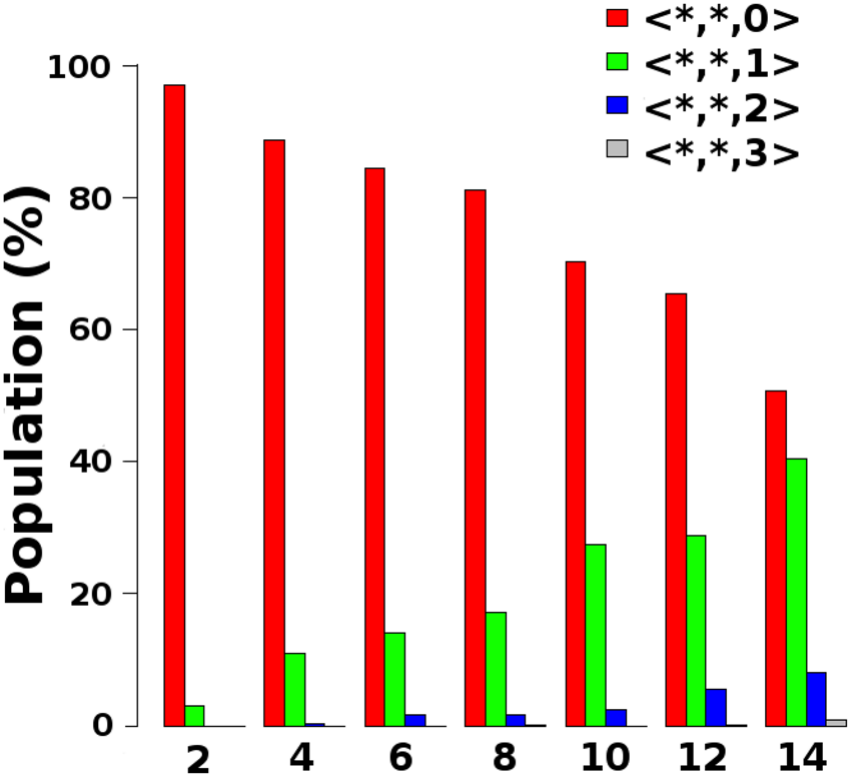
Relative populations of Al-centered clusters with different numbers of Al atoms in the first coordination shell, computed from CNA for each value of *x* in the series of BMGs ZCA-*x*. The respective populations are labeled according to the first coordination shell composition index, which was defined as 〈*n*_*Zr*_, *n*_*Cu*_, *n*_*Al*_〉, with *n*_*S*_ the number of first-neighbor atoms with symbol *S*.

The CNA offers an alternative way to group the Al-CCs based on their SRO. We started by using a cluster-type-index as described in ref. 26, which is suitable for highly distorted CPs and can reveal possible ambiguities from VA. However, even with this index it is still impossible to establish the correlations that we are looking for. Since all the analyses of the SRO considered so far were focused solely on configurational aspects of the FCS of the Al-CCs, we have chosen a straightforward FCS composition index (FCSCI) defined as 〈*n*_*Zr*_, *n*_*Cu*_, *n*_*Al*_〉, with *n*_*S*_ the number of first-neighbor atoms (according to the CNA) with symbol *S*. Once again, as can be seen in supplementary Figs S6 and S7, the scenario remains the same. However, if we group the Al-CCs according to the number of Al atoms existing on their FCSs, i.e., independent of *n*_*Zr*_ and *n*_*Cu*_ (the FCSCIs 〈*, *, *n*_*Al*_〉), it is possible to see in Fig. 3 a first characteristic of the FCS that appears to be associated with the ssNMR experiments under discussion. It may sound obvious, since the Al concentration is increasing in the BMGs ZCA-*x*. But makes evident that, in all nominal compositions, only few Al-CCs have more than one Al atom as a first-neighbor. So, up to this point, the SRO mining results confirms the conclusions made from Fig. 1 that Al atoms tend to cluster at distances exceeding the FCS, indicating that the composition/configuration of the first-neighbor atoms may not be the dominating factor determining the ^27^Al ssNMR metallic shifts measured for the concerned BMGs.

So far we have been able to identify the Al-CCs as primary coarse-grained entities, whose internal structures do not seem likely to explain by itself the influence of Al alloying on the experimental ^27^Al metallic shifts reported by Xi *et al*.^16^. Based on the first minima of the PRDFs shown in Fig. 1, these clusters can be assumed *quasi*-*equivalent* in terms of their volumes and, for high Al concentrations, the solute atoms tend to cluster whereas neighbor contact between them is avoided as much as possible. Actually, it is well known that exists a chemical affinity between Al, which is a metalloid in this context, and the transition metals (TMs) Zr and Cu in the BMGs under study^27^. Such chemistry defines a next structural level, characterized by superclusters of Al-CCs interconnected by shared atoms (common neighbors) in their respective FCS. The CNA divides the FCS of each atomic cluster in groups of atoms that share common neighbors. We took advantage of that intrinsic feature of this analysis to establish a criterion to define which atoms are shared by two linked Al-CCs. Firstly, through an index similar to the previously used FCSCI, we show in supplementary Figs S8 and S9 that the composition of the interconnection region between two linked Al-CCs, also cannot be used to explain the evolution of the experimental ^27^Al metallic shifts as the concentration of the solute specie Al increases.

**Figure 4:**
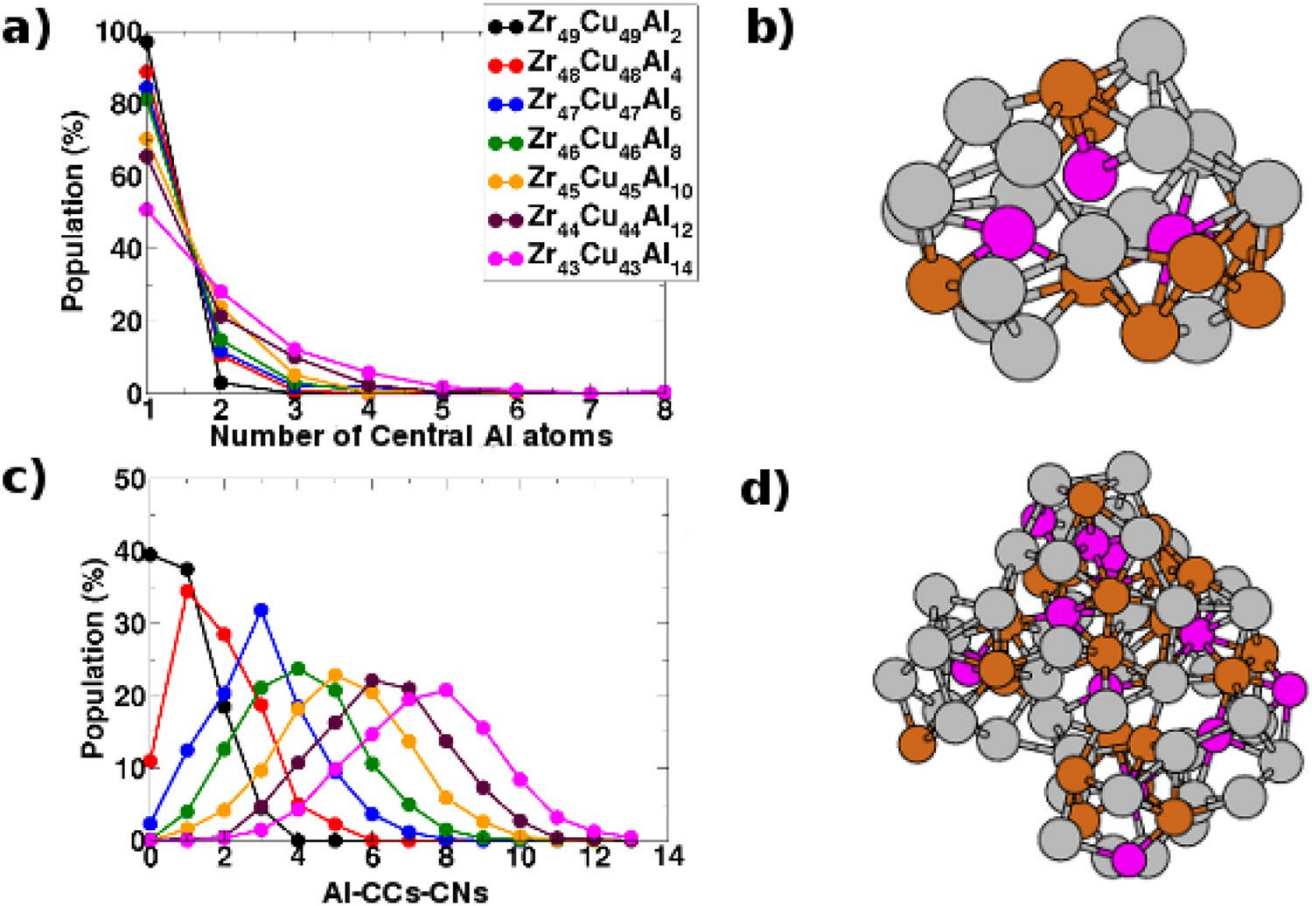
(**a**) Relative populations of extended clusters (see text and ref. 27) with different numbers of Al central atoms, computed from CNA for each value of *x* in the series of BMGs ZCA-*x*. The relative populations of non-extended clusters with a single Al central atom are also shown. (**b**) Example of an extended cluster with 3 central Al atoms. (**c**) Relative populations of Al-CCs coordination numbers (Al-CCs-CNs), as described in the text, computed from CNA for each value of *x* in the series of BMGs ZCA-*x*. (**d**) Example of a packing of 8 Al-CCs around a central Al-CC.

There are other types of MRO that may develop in BMGs^27–29^. We have found that the fraction of Al-CCs with one single first-neighbor Al atom increases upon alloying of this element (see Fig. 3). Therefore, we used the CNA to explore the extension and amount of possible extended clusters, which are nothing more than a type of MRO consisting of neighboring solute Al atoms forming chain-like structures surrounded by solvent Cu and Zr atoms^27^. We show in Fig. 4(a) that as the Al concentration increases, the extended clusters increase moderately in number and extension. But it is evident that the populations of these clusters with more than 2 central Al atoms, as in the example shown in Fig. 4(b) with 3 central Al atoms, are negligible. At least for the BMGs ZCA-*x* with *x* ≤ 10.

A last type of MRO considered by us comes from the concept of *quasi*-*equivalent* clusters described in ref. 27. As already mentioned, the Al-CCs like the ones depicted in Fig. 2(c,d) can be assumed *quasi*-*equivalent* in terms of their volumes and shapes. That type of MRO concerns the packing of such Al-centered *quasi*-*equivalent* clusters and, regardless the topological nature of the packing, we focus on the number of Al-CCs directly connected to a central Al-CC, hereinafter referred to as Al-CCs coordination numbers (Al-CCs-CNs). We show in Fig. 4(c) the distribution of the relative populations of these Al-CCs-CNs for each one of the BMGs ZCA-*x*. Also, we depict in Fig. 4(d) an example of a packing of 8 Al-CCs around a central one (Al-CCs-CN = 8). As can be seen in Fig. 4(c), this is the structural characteristic of the BMGs whose behavior upon Al alloying most closely resembles the convergence of the experimental metallic shifts reported in ref. 16.

The statistics from Figs 3 and 4 brings clear evidence of the strong chemical SRO favoring *unlike* Al-TM bonds, as discussed in ref. 27. These *unlike* bonds prevail over the *like* Al-Al bonds due to the aforementioned chemical affinity between Al and the TMs Zr and Cu in the BMGs ZCA-*x*. One can see in Fig. 4(c) that the population of isolated Al-CCs (Al-CCs-CN = 0) decreases sharply from *x* = 2 to *x* = 4, and that they virtually cease to exist for higher Al concentrations. Conversely, in Fig. 4(a), it can be seen that the populations of extended clusters with two central Al atoms start to rise very smoothly from *x* = 4. Such lack of direct Al-Al contacts was termed *solute*-*solute avoidance* in ref. 27, but their existence in the samples points to a sort of saturation of the chemical nature of the driven forces promoting the formation of *unlike* Al-TM bonds. This is compatible with the broadening of the Al-CCs-CNs populations distributions from *x* = 8 as shown in Fig. 4(c), barely reaching a maximum of Al-CCs-CN = 10 in the extrapolated concentration of *x* = 14. What is less than the 20 faces of a regular icosahedron.

The top issue is that the experimental metallic shifts reported for the BMGs ZCA-*x* in ref. 16 presented an exponential decrease from 320 ppm for *x* = 2, converging to about 280 ppm from *x* = 8 up to *x* = 10. This is a behavior that may be associated with the saturation described above and there must be a feature of the local/semi-local electron density around target Al sites that converges even for higher Al concentrations. So, before attempting to propose any mechanism based only on the CMD outcomes, in the next section we resort to first principles electronic structure simulations, guided by these trends describing changes on the microstructure of the material upon Al alloying that we have found so far.

### *Ab initio* simulations of *σ*_*o*_ and *σ*_*s*_ for abstract systems

From the order mining results presented in last section, we could identify trends from a perspective of the SRO and MRO around Al solute atoms. They certainly can be used alone to discuss the experimental ssNMR outcomes reported by Xi *et al*.^16^ for our case study BMGs. Nevertheless, it is important to be aware that ssNMR is not able to probe by itself their atomic structures upon Al alloying. In fact, in the specific case of those Al-TMs glassy alloys, the observed metallic shifts only point to concomitant changes in their local/semi-local atomic arrangement and bonding nature. Both aspects are relevant to understand the mechanical properties of such materials and, as already commented, ssNMR can be complemented by other experimental or theoretical techniques. Regarding the structural aspect, MD simulations is a well established aid, not only for glasses but even for complex biosystems^30^. From the electronic structure point of view in turn, EELS has been shown to be very efficient in offering experimental evidences of hybridization bonding mechanisms in Al-TMs BMGs, usually supported by *ab initio* DFT calculations^14^.

Our main objective in the present work is to show that substantial insights into the electronic structure of BMGs can be obtained from *ab initio* DFT simulations of density of states (DOS) and of ssNMR spectral parameters with the GIPAW method. In a recent report by one of us *et al*.^20^, this method was used successfully to quantify ^27^Al chemical and Knight shifts (CS and KS) isotropic values for a series of intermetallic compounds with formulas Sc*T*_2_Al (*T* = Ni, Pd, Pt, Cu, Ag, Au). We already stated that the computation of *σ*_*o*_ and *σ*_*s*_ components of the total shielding directly from the CMD derived large-scale structural models with 10000 atoms is not computationally feasible. Actually, as well covered in ref. 20, due to numerical convergence issues particularly for the *σ*_*o*_ component, the GIPAW computations are already quite expensive for structures with more than 20 atoms. Notwithstanding these remarks, and besides the aforementioned premise that ssNMR is intrinsically a short-ranged limited characterization technique, we present another argument to justify the use of abstract systems (ASs) to perform our *ab initio* DFT simulations. The ordinary ssNMR spectra of Al-TMs glassy alloys reported in the literature^13, 14, 17^ consist of a single distinguishable peak, which is usually broad with widths at half height of about 200 ppm. So, any attempts to correlate ssNMR data with properties of different BMGs reported so far, have been made by comparisons between their spectra. What is also valid for the experimental metallic shifts reported by Xi *et al*.^16^ for the BMGs ZCA-*x*. In such systems, the line broadening is normally ascribed to dipolar interactions, KS anisotropy or second order quadrupole couplings^31^. Recalling that the spin of ^27^Al is *I* = 5/2 and so, they are susceptible to quadrupole effect. Therefore, for a discussion of such experimental results, it is expected that the moderate precision and good accuracy achieved with the GIPAW method, demonstrated in ref. 20, is enough. Moreover, since it does not make much sense to explore the anisotropy of the nuclear interactions in non-realistic structures, we have chosen ASs with a minimal influence of quadrupole effect and KS anisotropy, i.e., supercells not only with an affordable number of atoms, but also with rather symmetric structures.

In short, the GIPAW method is used for the calculation of the orbital component (*σ*_*o*_) of the shielding tensor in metallic systems with a linear response formalism well described in ref. 32. In the case of the spin component (*σ*_*s*_), a more simple strategy is adopted and only the projector augmented waves (PAW)^33^ method is necessary for the all-electron wave functions reconstruction from their pseudo counterparts. These must have been computed previously in a plane wave (PW) pseudopotential approach. In a ssNMR experiment with a metallic non-magnetic material, the spin-degeneracy of the conduction electrons is broken in the presence of the uniform external magnetic field (**B**_*ext*_). In our DFT simulations (see Methods section for more details), this backdrop is mimicked by constraining the ground state spin-polarized electronic structure to converge with a total magnetization (*m*_*s*_), what is an input of the calculation. So, it is possible to obtain the spin-densities at the nuclear positions due to valence and core electrons (*ρ*_*s*_, see Eq. 1 in the Methods section), which are recovered with the PAW method and the perturbative approach proposed in ref. 34 for core polarization. In systems with high symmetry, only the Fermi contact contribution to the induced magnetic hyperfine field (**B**_*Fc*_) counts, since the dipolar term vanishes, and so the **B**_*Fc*_ is simply calculated from *ρ*_*s*_. Finally, if the dependence of **B**_*Fc*_ with **B**_*ext*_ is linear, it can be assumed that the spin component of the magnetic susceptibility is proportional to the induced spin moment, and therefore the approximation *σ*_*s*_ = −**B**_*Fc*_/**B**_*ext*_ is made.

A straightforward choice for a first AS is the crystalline phase of the ZCA alloy ZrCu_2_Al. Since it is also a *Heusler* phase like the intermetallic ScCu_2_Al studied in ref. 20, it is quite suitable for initial comparative tests. The reader is referred to the supplementary information for detailed results from these tests, and to the Methods section for technical details on our GIPAW simulations. It is especially important to remark that we are presenting here, in an unprecedented way, a quantitative estimate of the nature of the difference between the metallic shifts observed experimentally for pure Al and those of Al-TMs glassy alloys reported in the literature^13, 14, 16, 17^. The quantitative separation of the CS and KS contributions to metallic shifts in the BMGs at issue in the present study, can be roughly estimated from the *σ*_*o*_ and *σ*_*s*_ values computed for ZrCu_2_Al. And in fact, this is already a significant insight into the electronic structure of this type of material that cannot be be simply extracted from the low-resolution ordinary ssNMR spectra usually reported in the literature. It is possible to say a priori that the Al nuclei in the BMGs and in ZrCu_2_Al are in similar chemical environments, where the hyperfine exchange interactions of their nuclear spins with the conduction electrons are minimal. This becomes evident if one compares the small KSs computed for these structures with those simulated for metallic Al in ref. 20. Recalling that we want to discuss the experimental results reported by Xi *et al*.^16^, that the ^27^Al metallic shifts decrease as the concentration of the solute specie Al increases in the BMGs ZCA-*x*. So, in order to obtain further trends from *ab initio* DFT electronic structure simulations that could help us in that regard, we planned a set of ASs based on the order mining results presented in last section for these systems.

**Figure 5:**
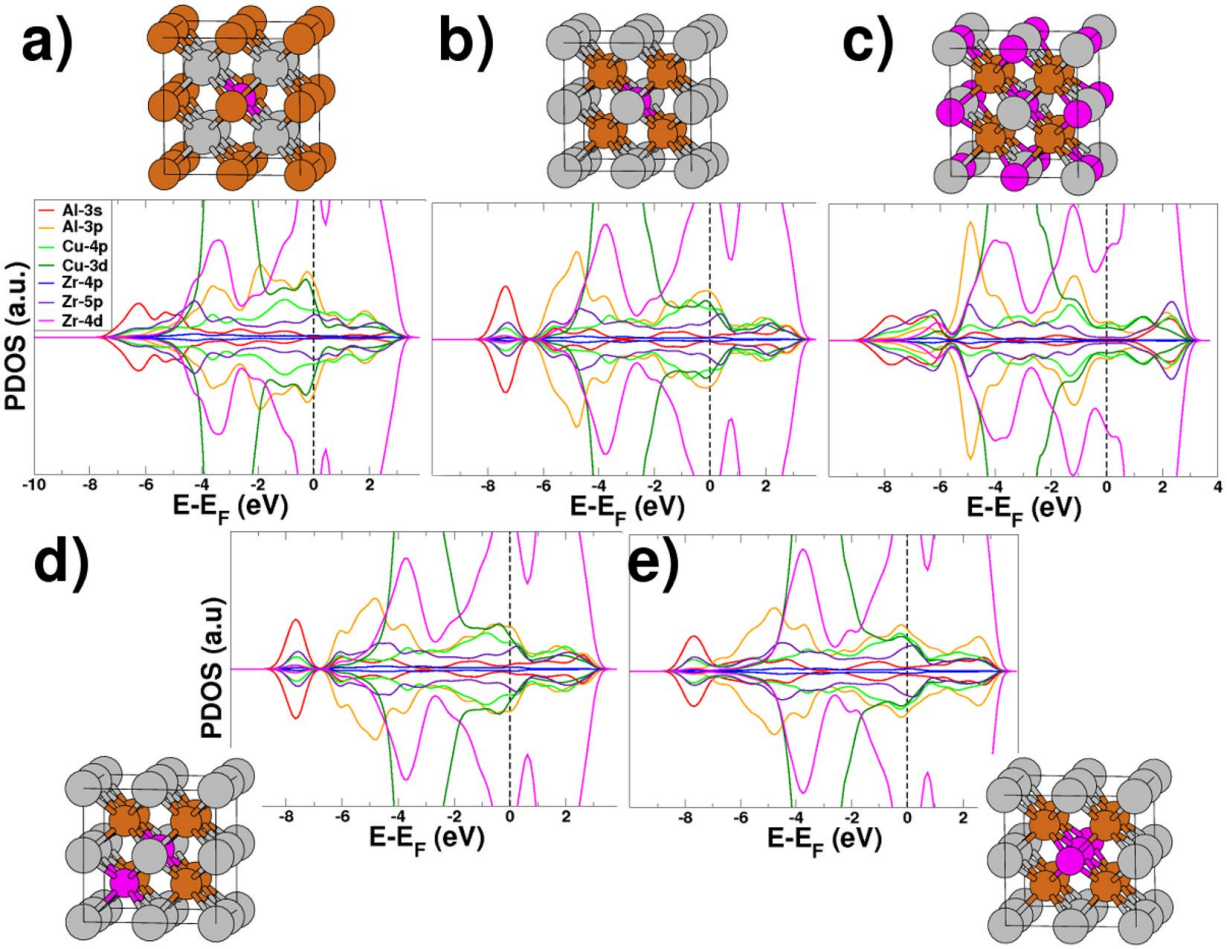
Projected density of states computed for the B2-CuZr derived abstract systems (**a**) B2-Cu_7_Zr_8_Al, (**b**) B2-Cu_8_Zr_7_Al, (**c**) B2-Cu_8_Zr_4_Al_4_, (**d**) B2-Cu_7_Zr_7_Al_2_-1^*st*^-Cu, and (**e**) B2-Cu_8_Zr_6_Al_2_-1^*st*^-Zr.

Initially, we follow the attempt of using DFT to discuss these same experimental results described by the authors of ref. 16 themselves, and start from B2-CuZr structure (with the $Pm\bar{3}m$ space group) to model the same two ASs named B2-Cu_8_Zr_7_Al and B2-Cu_7_Zr_8_Al. They were generated by replacing one Zr and one Cu by one Al atom in two distinct 2 × 2 × 2 supercells of B2-CuZr (containing 16 atoms each), as can be seen in supplementary Fig. S12. These two structures are also depicted in Fig. 5(a,b), which were both fully relaxed at the DFT level of theory without loss of the initial symmetry. The authors argued that these compositions are consistent with that of the BMGs ZCA-*x*. However, we point that, grounded on our previous CMD simulations, this is not true for the SRO and MRO around the Al sites.

So, based on our findings that the solute Al atoms tend to cluster whereas neighbor contact between them is avoided as much as possible, we proposed three additional ASs, which we call B2-Cu_8_Zr_6_Al_2_, B2-Cu_8_Zr_5_Al_3_, and B2-Cu_8_Zr_4_Al_4_. In other words, the building of this first set of ASs is guided by the packing of *quasi*-*equivalent* clusters associated to the *solute*-*solute avoidance* evidenced by the order mining with the CMD derived structures. These ASs were modeled from the structure of the AS B2-Cu_8_Zr_7_Al by replacing one of the Zr atoms 4.40 Å apart from the central Al-atom, by one Al atom as depicted in supplementary Fig. S12. Given the symmetry of that supercell, it can be seen as equivalent to gradually replacing 4 of the 12 Zr-centered rhombic dodecahedra (RD) connected to the central Al-centered RD. So, it is noticeable that the AS B2-Cu_8_Zr_4_Al_4_ is nothing more than the conventional crystallographic cell of the *Heusler* phase of ZrCu_2_Al. A summary of the metallic shifts related properties computed for all these ASs is given in Table 1. There, as commented in ref. 2, in the GIPAW formalism *σ*_*o*_ is determined primarily by the so called paramagnetic correction term (*σ*_Δ*p*_), which is strongly dependent on the chemical environment, i.e., on the Al-TM bonding nature. Here, we must point out that for all these bigger supercells, the *σ*_Δ*p*_ and *σ*_*o*_ were simulated without spin-polarization. After a simple test with ZrCu_2_Al, we found that these properties are rather insensitive to the small spin-degeneracy breaking imposed by the *m*_*s*_. In addition, these results obtained for the three B2-Cu_8_Zr_7_Al derived ASs were obtained without geometry relaxation. Since we are focused on trends of the isotropic *σ*_*s*_ values, we choose to preserve to the maximum the symmetry in these systems. We think that to explore the KS anisotropy in non-realistic structures would not yield useful results. Moreover, we underline that for the AS B2-Cu_8_Zr_4_Al_4_, which has a high symmetry, the *σ*_*s*_ value is 53.17 ppm in the optimized structure, which has a volume 10% lower than that of B2-Cu_8_Zr_7_Al.

**Table 1:** Isotropic components of the ssNMR spectral parameters and associated quantities computed for all the abstract systems, which are named with the prefix B2, followed by the number of Cu, Zr, and Al atoms, respectively.

	ZrCu_2_Al	B2-781	B2-871	B2-862	B2-853	B2-844	B2-772-1^*st*^-Cu	B2-862-1^*st*^-Zr
*m*_*s*_	0.6	25.1	30.5	49.8	22.8	3.0	19.2	37.4
${\rho }_{s}^{bare}$	−1	−12	−16	−25	−11	−2	−10	−19
${\rho }_{s}^{PAW}$	−6	−181	−142	−25	−10	0	−2	−139
${\rho }_{s}^{core}$	−7	−17	−13	8	11	−2	−12	−11
*ρ*_*s*_	−7	−210	−170	−41	−10	−4	−23	−169
*σ*_*s*_	53.17	1276.12	1291.31	311.43	86.81	30.38	174.71	1467.11
*σ*_Δ*p*_	−579.05	−499.90	−491.39	−436.37	−489.57	−522.82	n.c.	n.c.
*σ*_*o*_	182.21	294.36	272.25	327.01	269.84	230.21	n.c.	n.c.
*δ*_*iso*_	326.82	−1008.28	−1001.36	−76.24	205.55	301.61	n.c.	n.c.

See text for the meaning of the suffixes -1^*st*^-Cu and -1^*st*^-Zr. Chemical shieldings are given in parts per million (ppm) and spin-densities are in units of ${10}^{-6}{a}_{0}^{-3}$, with *a*_0_ the Bohr radius. The total magnetization (*m*_*s*_) values are given in units of 10^−3^ *μ*_*B*_, with *μ*_*B*_ the Bohr magneton. The isotropic chemical shifts (*δ*_*iso*_) were computed as described in the Methods section (see Eq. 2).

**Figure 6:**
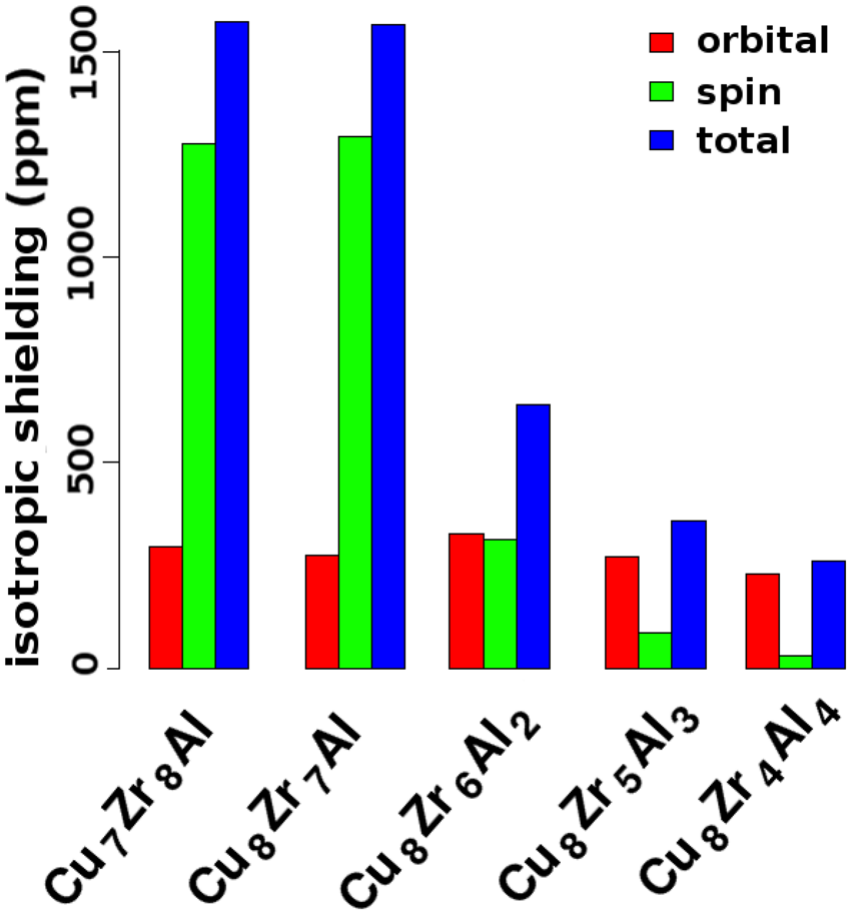
Isotropic shieldings computed for the B2-CuZr derived abstract systems.

The *σ*_*o*_ and *σ*_*s*_ values computed for all ASs considered so far in our work are graphed in Fig. 6, from which we highlight two main relationships between the SRO/MRO around the central Al sites and the respective ssNMR spectral parameters. Firstly, by comparing the simulations for the ASs B2-Cu_8_Zr_7_Al and B2-Cu_7_Zr_8_Al, it can be seen that in a same symmetric RD geometry, the metallic shifts are insensitive to the distribution of Zr and Cu atoms on the FCS. At this point, it is important to revisit the projected DOS (PDOS) analyses made by Xi *et al*.^16^ for these two same ASs. In that work, the authors justified the small ^27^Al metallic shifts observed in their ssNMR spectra obtained for the BMGs ZCA-*x*, with arguments essentially focused on Al-*3s* PDOS at the Fermi level (*g*_*s*_(*E*_*F*_)). As can be seen in Fig. 5(a,b), although the qualitative compatibility between our PDOS and theirs, all simulated with the same ASs, the respective *σ*_*o*_ and *σ*_*s*_ values in Fig. 6 show that the ^27^Al metallic shifts are as high as those found for pure Al metal in ref. 20. We ascribe such inconsistency in their analyses to two main factors. One is the well known inability of projection-based techniques, from a plane wave to an atom-centered basis set, to offer a precise description of the electronic structure in systems with essentially metallic bonds^35^. The other factor is that only pseudo-valence states have been taken into account in the PDOS plots. We believe that the use of the PAW/GIPAW approach from first principles provides a more concrete link between structure and metallic shifts, with essential accuracy and precision.

The other feature that is clear in Fig. 6 concerns how the computed *σ*_*o*_ and *σ*_*s*_ values behave as the number of Al atoms beyond the FCS of the central Al site increases in our set of ASs. Whereas *σ*_*o*_ values are virtually unaffected by such changes on the MRO around the central Al site, the response of *σ*_*s*_ is quite in line with the decrease in ^27^Al metallic shifts upon Al alloying reported by Xi *et al*.^16^. However, looking at the respective isotropic chemical shifts (*δ*_*iso*_), it becomes clear that the local/semi-local electron density around target Al sites in the ASs B2-Cu_8_Zr_7_Al and B2-Cu_7_Zr_8_Al are completely incompatible with the those on the real BMGs ZCA-*x*. Recalling that the experimental spectra are broad with widths at half height of about 200 ppm^13, 14, 17^, i.e., it would be possible to accept theoretical *δ*_*iso*_ values between 100 and 500 ppm. But even so, these two ASs which were also adopted in ref. 16, are definitely non-realistic. This is also true, albeit to a lesser extent, for the AS B2-Cu_8_Zr_6_Al_2_, whereas the other ASs yielded *δ*_*iso*_ values more compatible with the experiments. It is important to remark here that the lack of accuracy in these values can be solely assigned to the structural differences between the Al sites in the ASs and in the real materials. In a very recent publication^36^, the high precision of the GIPAW simulations reported for the intermetallic ScCu_2_Al in ref. 20 was confirmed, with the effectiveness of the well known fortuitous error cancellations in the computation of *δ*_*iso*_ values^37, 38^. Nevertheless, it must be stressed that the AS strategy presented here is essential, since the computation of NMR shifts in metals is still numerically challenging.

In any case, it is quite clear that with our initial set of ASs, it is possible to capture the key structural feature associated to the low metallic shifts observed in the experiments with ZCA BMGs. Revisiting Fig. 4(c), one can see that even for the lowest Al content in the BMG ZCA-2, the population of Al-CCs-CNs ≥1 is about 60%. Now, from Fig. S8, it is possible to verify that the vast majority of linked Al-CCs contain up to 3 Zr atoms in the respective interconnection regions. Despite present in the FCS of the Al-CCs, our statistics show that Cu atoms are not shared by linked Al-CCs. So, it is likely that the formation of ZrAl_*n*_ motifs is the referred key structural feature present not only in the ASs but also in the CMD derived structural models. Moreover, it is expected that the chemical aspect of such motifs that will affect the metallic shifts is a *spd* hybridization, what is well accepted for this type of materials^14, 39^.

There are some competing spin-polarization mechanisms normally evoked to explain the metallic shifts observed in Al-TMs BMGs, grounded on arguments associated to the chemistry of SRO and MRO around Al sites. From our order mining presented in last section, associated to the trend mentioned above and shown in Fig. 6, one can picture a sort of through-common-neighbor effect on the electronic structure of the interconnections regions between linked Al-CCs. Despite not precise enough to estimate quantitatively the metallic shifts, the PDOS analysis can offer a qualitative view of chemical bonding in our ASs. We show in Fig. 5, and also in Figs S13 and S14, the PDOS computed for our ASs, from which it is noticeable the essential delocalization of energy states characteristic of metallic systems. Nonetheless, the partial covalent-like bonding character introduced by the *spd* hybridization between the metalloid Al and the TMs is apparent as well. Before proceeding the discussion, we point out two technical details regarding these results. First, given their high symmetry structures, all the Zr and Cu atoms which are equivalent by symmetry have virtually the same PDOS and so, we are considering only those in the FCS of the respective central Al site. As last remark is that only the pseudo electronic states, expanded in the PWs basis, are being projected.

The hybridization bonding mechanisms and the *g*_*s*_(*E*_*F*_) have been the main focus of discussion in works where PDOS from first principles DFT calculations, or experiments like ssNMR or EELS, are used to probe the electronic structure of Al-TMs BMGs^13, 14, 16, 23^. It is well accepted that the small metallic shifts observed in their spectra are mainly due to low *g*_*s*_(*E*_*F*_), what is in good agreement with our PDOS for all ASs. However, this is not compatible with their respective *σ*_*s*_ values listed in Table 1. It is even possible to notice small differences, if we compare the *g*_*s*_(*E*_*F*_) computed for the series of ASs B2-Cu_8_Zr_(8−*x*)_Al_*x*_. However, this is not the only feature of their PDOS that can be explored to discuss the effect of the alloying element Al on the local/semi-local electronic structure around the preexisting Al-CCs.

In our ASs, different degrees of hybridization among Al-*3s*, Al-*3p*, Zr-*4d*, and Cu-*3d* bands, or only between Zr-*4d* and Cu-*3d*, are evident in all PDOS plots in Fig. 5, at energies around −4.0 and −0.2 eV. Different degrees of degeneracy of the Al-*3p* orbital are also noticeable in each PDOS, with two bonded states located at these two energy levels. In the AS B2-Cu_7_Zr_8_Al, it is noticeable a non-bonded Al-*3p* orbital at around −2.0 eV whereas in the series of ASs B2-Cu_8_Zr_(8−*x*)_Al_*x*_, its distribution covers a range between −6.5 and −4.5 eV, which becomes narrower and centered at around −5.0 eV as the Al concentration increases. Following the Al addition by removing Zr atoms in that series of ASs, two regions of their respective PDOS spectra change prominently. The first is exactly at the Fermi level. The high overlap of Al-*3p*, Zr-*4d*, and Cu-*3d* states tends to shift toward low energy values upon that structural change, strengthening the Al-Cu and Al-Zr chemical bonds. But the most important feature of the AS B2-Cu_8_Zr_4_Al_4_ PDOS is the pseudogap-like electronic structure near the Fermi level, which was also proposed for the BMGs Zr_60_ *TM*_28_Al_12_ (*TM* = Co, Ni, and Cu) by Yuan *et al*.^14^, based on their EELS experiments. As well as in our simulations, the decreasing of *d* orbitals partially occupied is reflected in their respective PDOS at the Fermi level, *g*_*d*_(*E*_*F*_). In that work, the authors also observed that the experimental ^27^Al metallic shifts of those BMGs decrease following the order *TM* = Co, Ni, and Cu, what is also true for the respective magnitudes of *g*_*d*_(*E*_*F*_) estimated from EELS experiments. Based on that correlation, an exchange core polarization mechanism was assumed to justify the outcomes of their ssNMR experiments.

We remark that *g*_*d*_(*E*_*F*_) is one of the features of our PDOS that is most likely to explain the *σ*_*s*_ values computed for our ASs. Moreover, the respective ${\rho }_{s}^{core}$ magnitudes are also in qualitative agreement with that mechanism. However, although reinforce the relevance of core-polarization in PAW calculations of hyperfine parameters, it is clear from Table 1 that the spin-densities at the nuclear positions due to valence electrons ${\rho }_{s}^{val}={\rho }_{s}^{bare}+{\rho }_{s}^{PAW}$ (see Eq. 1 in the Methods section), are the most expressive components of *ρ*_*s*_. Actually, the PAW related densities, ${\rho }_{s}^{PAW}$, are the portions that dictate the *σ*_*s*_ values in our ASs. Since only the reconstruction terms corresponding to *s* states contribute to ${\rho }_{s}^{PAW}$, this is an indication that the *g*_*s*_(*E*_*F*_) prevails over the core-polarization mechanism due to the *g*_*d*_(*E*_*F*_) in the ASs B2-Cu_7_Zr_8_Al and B2-Cu_8_Zr_7_Al, for which we computed *σ*_*s*_ values as high as those found for pure Al metal (see ref. 20). As we have already commented, the analyses of the PDOS from pseudo valence states is not able to reveal such details of the electronic structure in these systems. By contrast, the PAW method allows the recovery of the nodal structure of the valence states, what is fundamental for the calculation of hyperfine parameters with accuracy. It can be checked in ref. 20 that this is also valid for metallic Al. With respect to the other ASs with low *σ*_*s*_ values, the effect of *g*_*s*_(*E*_*F*_) and of the core-polarization mechanism from *g*_*d*_(*E*_*F*_) are more balanced, as can be verified in Table 1.

That prevalence of the *g*_*s*_(*E*_*F*_) over the core-polarization mechanism due to the *g*_*d*_(*E*_*F*_), is not apparent in the PDOS plots for the ASs B2-Cu_7_Zr_8_Al and B2-Cu_8_Zr_7_Al. However, there is another region of the PDOS spectra of all ASs that changes gradually as *x* increases in the series of ASs B2-Cu_8_Zr_(8−*x*)_Al_*x*_. The range from from −8.0 to −6.0 eV, is where the PDOS of the a priori non-hybridized Al-*3s* orbitals are concentrated. This is notorious in the PDOS of the ASs with *σ*_*s*_ values in excess of 1000 ppm, pointing to a strong spatial localization of these states around the Al site. Particularly for the AS B2-Cu_8_Zr_7_Al, it is possible to notice the gap in the valence bands at −6.5 eV already described in earlier studies with Al containing metallic glasses^40^. So, the gradual formation of ZrAl_*n*_ motifs in the in the series of ASs B2-Cu_8_Zr_(8−*x*)_Al_*x*_, although not absolutely accurate, introduces a spreading of the Al-*3s* PDOS up to −6.0 eV with a mixture with other states, including the Al-*3p*. Again, it is really important to highlight that the effect of such hybridization on the *g*_*s*_(*E*_*F*_) at Al sites only can be captured with the PAW reconstruction. A bare analysis of the PDOS plots of pseudo states cannot provide a visual evidence of that.

Finally, we recall that the order mining results presented in last section pointed to a number of Al atoms existing in extended clusters (see Fig. 4(a,b)). So, one could picture a sort of through-bond effect that could also influence the ^27^Al metallic shifts of directly bonded Al atoms. In order to check it out, we also considered two additional ASs created from the B2-Cu_8_Zr_7_Al structure by replacing one Cu and one Zr atom at the FCS of the central Al atom by an additional Al atom, in each AS. We named these ASs B2-Cu_7_Zr_7_Al_2_-1^*st*^-Cu and B2-Cu_8_Zr_6_Al_2_-1^*st*^-Zr, respectively, whose PDOS and supercells are depicted in Fig. 5(d,e). Their respective ^27^Al metallic shifts related quantities are also listed in Table 1 and it is possible to see that the same conclusions made for the other ASs are also valid for them. A last addendum concerns the KS anisotropy verified in our ASs. The ASs B2-Cu_8_Zr_7_Al, B2-Cu_7_Zr_8_Al, and B2-Cu_8_Zr_4_Al_4_ are high symmetric and the eigenvalues KS anisotropy tensor are all equals to zero. On the other hand, relatively small eigenvalues were computed for the other ASs. At any rate, we already pointed that such non-realistic structures are not suitable to explore neither the KS anisotropy nor the quadrupole interactions in BMGs.

## Conclusion

Through a consistent combination of classical molecular dynamics (CMD) and DFT simulations, the experimental ^27^Al ssNMR metallic shifts of ZCA BMGs were scrutinized in an unprecedented manner. The ssNMR experiments reported by Xi *et al*.^16^ for a set of BMGs with nominal compositions Zr_(50−0.5*x*)_Cu_(50−0.5*x*)_Al_*x*_, with *x* = 2, 4, 6, 8, and 10, were selected as case studies. From their respective realistic CMD derived structural models, a data mining procedure was applied with the focus on the SRO and MRO around the Al atoms. Statistical results pointed to trends that describe how do the solute atoms cluster upon Al alloying at a fixed Zr/Cu ratio. In summary, the packing of Al-centered *quasi*-*equivalent* clusters (Al-CCs) is ruled by a strong chemical SRO favoring *unlike* Al-TM bonds, and also by a *solute*-*solute avoidance* that result in a lack of Al-Al contacts. Besides the Al-CCs themselves, the key MRO structural feature of these BMGs is the formation of ZrAl_*n*_ motifs, with Zr atoms playing a central role of linkers among the packed Al-CCs, whereas Cu atoms are not shared by them. Naturally, that information could already be useful to justify the metallic shifts at issue. However, instead, these structural trends were used to guide a DFT/GIPAW study to compute first principles PDOS and ssNMR spectral parameters for a series of ASs. They are abstract in the sense that the main structural characteristics revealed by the SRO and MRO mining could be gradually reproduced with affordable structural models. What is in line with the short-ranged structural information obtained from ssNMR experiments. Although not absolutely accurate in terms of structure, the *ab initio* simulations with the ASs revealed significant insights into the bonding states of the BMGs under study. They showed that the formation of ZrAl_*n*_ motifs linking the packed Al-CCs, found in the CMD derived systems, is the main structural feature associated to the small ^27^Al metallic shifts observed in experiments. Furthermore, they suggested that the hybridization between Al-*3p* and Zr-*4d* orbitals is a relevant aspect of the local/semi-local electron density around target Al sites that converges even for higher Al concentrations. From the electronic structure point of view, a first and dominating spin-polarization mechanism is related to the hybridization of Al-*3s* orbitals with other states, what does not take place in pure Al metal and decreases the density of these states at the Fermi level. Additionally, a second mechanism, which is of an exchange core polarization nature, comes from the formation of a pseudogap due to a decrease in the density of Zr-*4d* states at the Fermi level.

The challenging task of estimate the competing CS and KS contributions to the experimental ssNMR metallic shifts in BMGs, is motivated by the possibility of probing the electronic structure at specific sites. That represents valuable information which can promote deeper understanding of the mechanical properties of these materials. Although ssNMR is not able to probe the SRO and MRO in BMGs by itself, it has been shown in the literature that, in the specific case of Al-TMs glassy alloys, supporting experimental techniques like spin-lattice relaxation times^16^ and EELS^14^ can be used to investigate the bonding nature of Al-CCs. The first principles PDOS and GIPAW results, more specifically the *ρ*_*s*_ components that define *σ*_*s*_ values, provided quantitative predictions with essential precision and accuracy that can be used to corroborate the interpretation of such experiments. Or even to replace them as complementary techniques in the interpretation of ssNMR outcomes. Moreover, the same CMD structural models of BMGs used for the SRO and MRO mining, can also be used to simulate a large variety of mechanical properties. So, an approach similar to the one exposed in the present work, can offer detailed theoretical assessment, from an atomistic/electronic structure point of view, of the relationships between the mechanical behavior of BMGs with experimental ssNMR metallic shifts, as reported in recent studies^13, 17^. Despite the necessity of further developments concerning the simulations of ssNMR spectral parameters for metallic systems with the GIPAW method as discussed in ref. 20, the results presented here demonstrate the possibility to expand the applications of ssNMR spectroscopy, with deeper insight into nuclear interactions and less speculative assignments.

## Methods

### CMD simulations

All the CMD simulations were carried out using the Velocity Verlet algorithm as implemented in the LAMMPS package^41^. The EAM potential developed and validated extensively by Cheng *et al*.^23^ was used with a cutoff radius of 6.5 Å to describe the interatomic forces in all the BMGs with nominal compositions Zr_47_Cu_46_Al_7_ and Zr_(50−0.5*x*)_Cu_(50−0.5*x*)_Al_*x*_, with *x* = 2, 4, 6, 8, 10, 12, and 14. More precisely, the EAM potential made available on October 10, 2011 in ref. 24 was used. A same procedure was followed to obtain their respective structural models. An initial configuration was set by randomly positioning 10000 atoms in cubic supercells. The initial volumes were estimated from each BMG nominal composition, initially assuming a dense sphere packing weighted with the Zr, Cu, and Al atomic radii and then adding a length in excess of 10 Å for each box. In order to avoid superposition of atoms, we executed a conjugate gradient minimization on the random initial structure with an unique stop criterion defined by a force threshold of 10^−8^ eV/Å. Next, the system was thermalized at 2000 K in the *NPT* ensemble for Δ*t* = 2 ns, using the Nosé-Hoover thermostat with a dump coefficient of 0.2 ps and a barostat set to zero pressure with a dump coefficient of 2 ps. Subsequently, the system was cooled to 300 K with a cooling rate selected from the already discussed initial tests shown in Table S1. Finally, a second thermalization at 300 K for Δ*t* = 0.02 ns was performed before the structural analyses. We also point that a time step of 2 fs was adopted for all simulations, after a set of tests made with the system Zr_47_Cu_46_Al_7_ and a CR of 8.5 × 10^12^ K/s, considering time steps between 1 to 5 fs. We found that time steps exceeding 3 fs brought numerical instabilities to our simulations, mainly at 2000 K. Also, the Voronoi and common neighbor analyses (CNA) were made as described in the text with an addendum that a CNA cutoff radius of 3.75 Å was chosen according to the first minima of the partial radial distribution functions shown in Fig. 1.

### *Ab initio* electronic structure simulations

All the PDOS and GIPAW results were obtained from electronic structure simulations performed using the Quantum ESPRESSO^42^ (QE) open-source software suite version 5.3.0, which offers an implementation of the DFT^6, 7^ with PWs and pseudopotentials or PAW^33^ datasets. As mentioned in ref. 20, the GIPAW calculations were carried out with the QE-GIPAW module SVN revision 408. Which required minor adaptations for the computation of the orbital component (*σ*_*o*_) of the shielding tensor in spin-polarized metallic systems, with a linear response formalism well described in ref. 32. The spin component (*σ*_*s*_) of the shielding tensor requires the computation of the spin-densities, which are given by the difference *ρ*_*s*_ = *ρ* ^↑^ − *ρ* ^↓^. We point that the electron density for spin channel *ξ* (↑ or ↓) at a position **r** in the PAW dataset radial grid, had already been implemented in that distribution of the QE-GIPAW module and is a sum of valence (the three first terms in the right hand side of the equation below) and core contributions

1 $${\rho }^{\xi }({\bf{r}})={\tilde{\rho }}^{\xi }({\bf{r}})+{\rho }_{1}^{\xi }({\bf{r}})-{\tilde{\rho }}_{1}^{\xi }({\bf{r}})+{\rho }^{c\xi }({\bf{r}}\mathrm{).}$$

where ${\tilde{\rho }}^{\xi }({\bf{r}})$ is the so-called bare contribution that depends on the variational functions $|{\tilde{{\rm{\Psi }}}}_{n,{\bf{k}}}^{\mathrm{(0)}}\rangle$, whilst ${\rho }_{1}^{\xi }({\bf{r}})$ and ${\tilde{\rho }}_{1}^{\xi }({\bf{r}})$ are the PAW one center reconstruction terms. The core contribution *ρ* ^*cξ*^(**r**) is with computed perturbative approach as described in ref. 34. The isotropic chemical shifts (*δ*_*iso*_) were computed as

2 $${\delta }_{iso}=({\sigma }_{ref}+{\delta }_{ref}^{exp})-({\sigma }_{o}+{\sigma }_{s}).$$

Here, aluminum phosphate, AlPO_4_, was adopted as a reference compound, for which we computed an isotropic chemical shielding *σ*_*ref*_ = 517.2 ppm. The value ${\delta }_{ref}^{exp}$ = 45 ppm, also used in refs 32 and 36, is its experimental shift with respect to aluminum chloride, AlCl_3_, in heavy water. A detailed description of all technicalities and theories related to the simulations of ssNMR spectral parameters for metallic systems can also be found in ref. 20.

The Perdew-Burke-Ernzerhof^43^ generalized gradient approximation was used to describe the exchange-correlation functional in all computations. All the PAW datasets considered in that last cited work have also been taken into account by us. However, given the relatively low level of precision required to discuss the experiments covered in our work, we verified, after some tests, that the PSLibrary project version 1.0.0 provided by Dal Corso^44^ could offer adequate accuracy. The Monkhorst-Pack procedure^45^ was used to determine, through systematic tests, the k-points samplings in the first Brillouin zone necessary to converge all the concerned properties for each system. A Fermi-Dirac probability distribution is used as a smearing function to set the occupations of energy levels for all ASs, with a common broadening parameter *k*_*b*_ *T* = 8 mRy. Also for all ASs, the PWs basis set was truncated to include only PWs with kinetic energies smaller than 80 Ry. Finally, the ground state spin-polarized electronic structure was converged for each system under the constraint of a total magnetization, which was set in such a way to mimick the respective induced spin magnetic moments caused by an external magnetic field of 7.01 T, the same used in the experiments of Xi *et al*.^16^. The number of k-points used to reach essential convergence of *σ*_*o*_ and *σ*_*s*_ values for all ASs are as follows: B2-Cu_7_Zr_8_Al (120 k-points); B2-Cu_8_Zr_7_Al (120 k-points); B2-Cu_8_Zr_6_Al_2_ (196 k-points); B2-Cu_8_Zr_5_Al_3_ (196 k-points); B2-Cu_8_Zr_4_Al_4_ (120 k-points); B2-Cu_7_Zr_7_Al_2_-1^*st*^-Cu (280 k-points); B2-Cu_8_Zr_6_Al_2_-1^*st*^-Zr (288 k-points).

## Electronic supplementary material

Supplementary Information
