# Supplementary material for: On the use of atomistic simulations to aid bulk metallic glasses structural elucidation with solid-state NMR
Source: Sci Rep. 2017 Aug 24;7:9305. doi: 10.1038/s41598-017-08919-6 (PMC5571217; doi:10.1038/s41598-017-08919-6)
Supplement: Supplementary file 1 — Supplementary Information [file 41598_2017_8919_MOESM1_ESM.doc]

**Supplementary information**

**On the use of atomistic simulations to aid bulk metallic glasses structural elucidation with solid-state NMR**

Ary R. Ferreira and José P. Rino^*^

*Department of Physics, Universidade Federal de São Carlos (UFSCar)*

*São Carlos–SP, 13565–905, Brazil*

^*^Corresponding author: djpr@df.ufscar.br

**1 – Initial tests with the BMG Zr_47_Cu_46_Al_7_**

As aforementioned, high cooling rates (CRs) of 10^4^ to 10^6^ K/s may be necessary to produce bulk metallic glasses (BMGs) but depending on the composition, critical CRs as low as 1 K/s have also been reported.**^1^** Moreover, in the specific case of the ternary Zr-Cu-Al (ZCA) alloy system, whose high glass forming ability is well known, a series of glassy samples with different nominal compositions have already been fabricated with a very low quenching rate of 1.67×10^-3^ K/s.**^2^** The top issue is that such slow cooling is not viable in common computational simulations, for which ultrafast quenching with rates of the order of 10^10^ to 10^12^ K/s are more frequently found in the literature. Naturally, it is expected that this discrepancy could well result in proportional poor outcomes from the simulations, due to the huge difference between the relaxation time available for the theoretical structural model and the real-world BMG structure.**^3^** However, it must be pointed that, contrary to MD simulations, slow cooling consist of an inherent limitation of the experiments, whose CRs values shall not be less than the critical CRs of the glassy alloy systems. In any case, from the theoretical point of view, we believe that it is worth exploring the behavior of the properties under study as the CR decreases.

Based on the assumption stated above, we assumed a minimal and feasible CR of 8.5×10^9^ K/s, equivalent to quench the supercooled liquid from 2000 K to 300 K for 200 ns in the time evolution of the MD simulation (Δt). Then, we further explore the effect of faster quenching on some ordinary properties of the BMG Zr_47_Cu_46_Al_7_. For technical details on our classical molecular dynamics (CMD) simulations, the reader is referred to the Methods section in the article. From Table S1, one can notice a sort of convergent behavior in the computed glass transition temperatures (T_g_) and equilibrium mass density at 300 K (ρ_m_^300 K^) values as the CR decreases. This behavior can be confirmed by inspecting the respective volume versus temperature curves in Fig. S1. From which it is also possible to verify the glass transition (a second order phase transition) for all CRs, characterized by the absence of any marked discontinuity in the curves. We also point that the theoretical T_g_ values computed from the simulations with low CRs are more compatible with the experimental counterparts reported by Yokoyama *et al.***^2^** for a series of ZCA alloys. Especially for the BMG Zr_50_Cu_42.5_Al_7.5_ (T_g_ = 700 K), whose nominal composition is closer to the system Zr_47_Cu_46_Al_7_. Our simulations are also consistent with the expectations that lower CRs would promote structural relaxation making the T_g_ decrease. Additionally, it should be mentioned that the ρ_m_^300 K^ values listed in Table S1 are i good agreement with the experimental 7.07 g/cc also reported by Cheng *et al.***^4^** for that same BMG.

The huge difference between the relaxation times experienced by a real BMG and by a CMD derived structural model after reach the room temperature, make the last be inevitably more strained and less ordered than the former. Nevertheless, a look at the partial radial distribution functions (PRDFs) in Fig. S2, computed for the four CMD derived structures of the BMG Zr_47_Cu_46_Al_7_ listed in Table S1, does not reveals a remarkably impact of the CR on the short- and medium-range order (SRO and MRO). So, in order to obtain further insight into the SRO and try to identify any evidence of structural relaxation right after quenching, we resorted to the Voronoi analysis (VA) method (see more in Ref. 5). For each CR, the VAs were repeated every Δt = 0.02 ps over the first Δt = 0.2 ps of the second thermalization at 300 K, which started immediately after the end of the cooling process. Recalling that the first thermalization was at 2000 K, before cooling, as described in the Methods section.

A readily available structural information from a VA is the coordination number (CN) of each atom in the CMD derived structural models. Our focus are the Al-centered clusters (Al-CCs) and we found a distribution of CNs between 10 and 17 for them, with CN = 12, 13, and 14 consisting of about 90% of all Al-CCs in each VA. This is is agreement with the integrals of the first peaks of the PRDFs (see Fig. S2) computed for the pairs Al-Zr, Al-Cu, and Al-Al. We show in Fig. S3 that the amount of Al-CCs with CN = 12, 13, and 14 (which we will label Al-CCs-12, Al-CCs-13, and Al-CCs-14, respectively from here) after the second thermalization at 300 K are affected by the different CRs. Moreover, it can be seen in Fig. S4 that a sort of structural relaxation is taking place, where time evolutions of different CNs show up as interconversion patterns, which are affected by the distinct CRs. Since relaxation in this type of system takes place through atoms migrations, a mechanism for such interconversions should consist of displacements that exceed the vibrations of Zr and Cu atoms in the first coordination sphere (FCS) of Al-CCs, resulting in jumps between neighbor clusters. Regardless of the differences in the compositions and geometries of the Al-CCs's FCS, it is clear that the dynamic equilibrium is reached at the first steps of the second thermalization at 300 K for the lowest CRs. In addition, it seems that the amounts of Al-CCs-12, Al-CCs-13, and Al-CCs-14 go to converge as the CR decreases. Therefore, from these first tests with the system Zr_47_Cu_46_Al_7_, we justify the further use of a CR = 8.5×10^9^ K/s.

**2 – Tests with the ZrCu_2_Al *Heusler* compound**

Here we present some initial tests related to the first principles simulations of the solid state nuclear magnetic resonance (ssNMR) spectral parameters. For technical details on our Gauge-Including Projector Augmented-Wave (GIPAW) simulations, the reader is referred to the Methods section in the article.

A straightforward choice for a first abstract system (AS) is the crystalline phase of the ZCA alloy ZrCu_2_Al. Since it is also a *Heusler* phase like the intermetallic ScCu_2_Al studied in Ref. 6, it is quite suitable for initial comparative tests. The first step was to ensure whether the relationship between the induced magnetic hyperfine field (**B**_Fc_) and the uniform external magnetic field (**B**_ext_) is linear for this system, what is confirmed by the plot in supplementary Fig. S10. As can be seen in that plot that, despite the intensity of the electron spin susceptibility be comparable to that computed for ScCu_2_Al, the linear dependence is inverse. This is certainly due to different spin polarization mechanisms in both compounds and is not relevant for now. In a next step, we investigate the effect of soft changes on the SRO around the only type of Al site existing in the ZrCu_2_Al structure, on the respective isotropic orbital (σ_o_) and spin (σ_s_) shielding values. It was done simply by varying isotropically the volume of its primitive cell, which contains only 4 atoms, as can be seen in supplementary Fig. S11(a). It amounts to change isotropically the volume of the rhombic dodecahedron (RD) coordination polyhedron (CP) shown in Fig. 1(d) (see article) and it can be seen in Fig. S11 that, for volumes around the DFT equilibrium lattice parameters (ΔV = ± 2%), the σ_s_ isotropic values vary proportionally with the atomic distances in a range between about 50 and 75 ppm. Moreover, it is remarkable that the σ_o_ isotropic values are insensitive to these variations. As commented in Ref. 7, in the GIPAW formalism σ_o_ is determined primarily by the so called paramagnetic correction term (σ_Δp_), which is strongly dependent on the chemical environment, i.e., on the Al-TM bonding nature. Based on the value of σ_Δp_ = -579.05 ppm computed for the ^27^Al nucleus in ZrCu_2_Al (see Table 3 in the article), and on those reported previously in Ref. 6 for the Sc*T*_2_Al compounds and also for metallic Al, it is possible to point a certain similarity between the chemical environment of Al in ZrCu_2_Al and in ScCu_2_Al. And this is also true for all components of the respective values computed for the spin-densities at the nuclear positions due to valence and core electrons (ρ_s_ = ρ_val_ + ρ_core_). Additionally, it is especially important to remark that we are presenting here, in an unprecedented way, a quantitative estimate of the nature of the difference between the metallic shifts observed experimentally for pure Al and those of Al-TMs glassy alloys reported in the literature.**^8-12^**

**3 – References**

1 - Busch, R., Schroers, J. & Wang, W. H. Thermodynamics and kinetics of bulk metallic glass. *MRSBulletin* **32**, 620–623 (2007). DOI 10.1557/mrs2007.122.

2 - Yokoyama, Y., Yamasaki, T., Liaw, P. K. & Inoue, A. Relations between the thermal and mechanical properties of cast zr-tm- al (tm: Cu, ni, or co) bulk glassy alloys. *Mater. Transactions* **48**, 1846–1849 (2007). DOI 10.2320/matertrans.MJ200717.

3 - Ding, J. & Ma, E. Computational modeling sheds light on structural evolution in metallic glasses and supercooled liquids. *NPJ Comp. Mater.* **3**, 9(1–12) (2017). DOI 10.1038/s41524-017-0007-1.

4 - Cheng, Y. Q., Ma, E. & Sheng, H. W. Atomic level structure in multicomponent bulk metallic glass. *Phys. Rev. Lett.* **102**, 245501(1–4) (2009). DOI 10.1103/PhysRevLett.102.245501

5 - Wey, Y. D. & Peng, P. A comparative study on local atomic configurations characterized by cluster-type-index method and voronoi polyhedron method. *Comp. Mater. Sci.* **123**, 214–223 (2016). DOI 10.1016/j.commatsci.2016.06.030.

6 - Ferreira, A. R., Reuter, K. & Scheurer, C. Toward routine gauge-including projector augmented-wave calculations for metallic systems: The case of sct 2 al (t = ni, pd, pt, cu, ag, au) heusler phases. *J. Phys. Chem. C* **120**, 25530–25540 (2016). DOI 10.1021/acs.jpcc.6b08418.

7 - Pickard, C. J. & Mauri, F. All-electron magnetic response with pseudopotentials: Nmr chemical shifts. *Phys. Rev. B* **63**, 245101–245113 (2001). DOI 10.1103/PhysRevB.63.245101.

8 - Xi, X. K. et al. Bonding characters of al-containing bulk metallic glasses studied by 27 al nmr. *J. Phys.: Condens. Matter* **23**, 115501(1–5) (2011). DOI 10.1088/0953-8984/23/11/115501.

9 - Yuan, C. C., Xiang, J. F., Xi, X. K. & Wang, W. H. Nmr signature of evolution of ductile-to-brittle transition in bulk metallic glasses. *Phys. Rev. Lett.* **107**, 236403(1–5) (2011). DOI 10.1103/PhysRevLett.107.236403.

10 - Sandor, M., Kecskes, L., He, Q., Xu, J. & Wu, Y. Correlation of mechanical properties in bulk metallic glasses with 27 al nmr characteristics. *Chin. Sci. Bull.* **56**, 3937–3941 (2011). DOI 10.1007/s11434-011-4834-z.

11 - Yuan, C. C. et al. Atomic and electronic structures of zr-(co,ni,cu)-al metallic glasses. *Appl. Phys. Lett.* **101**, 021902(1–4) (2012). DOI 10.1063/1.4734390.

**4 – Figures and Tables**

**Supplementary Figure S1**: The cooling curves for the BMG Zr_47_Cu_46_Al_7_ prepared via CMD simulations with different cooling rates (CRs). Each one of the five glass transition temperatures (*T_g_*) in the box at the bottom right corner were computed from the intersection of the glassy state and supercooled liquid cooling curves. A simple linear regression method was used and for each CR and two arbitrary ranges of points were selected, one above and other below a *T_g_* chosen by visual analysis of the plots. The respective *T_g_* values are listed on the right-down corner.


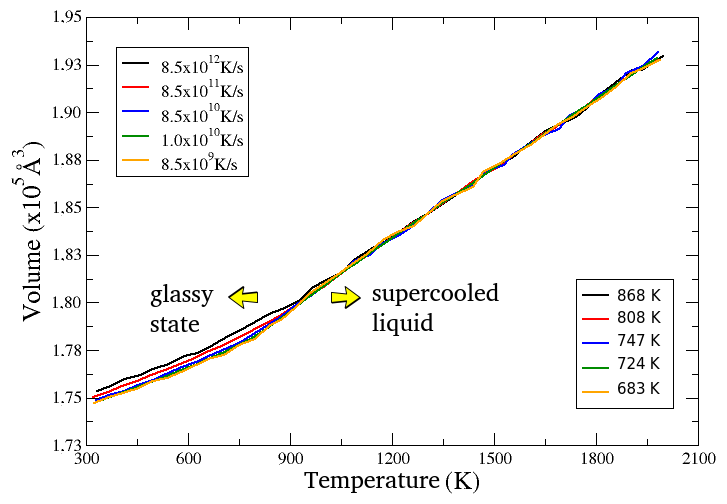


**Supplementary Figure S2**: Partial radial distribution functions (PRDFs) computed for the BMG Zr_47_Cu_46_Al_7_, prepared via CMD simulations with different cooling rates. The PRDFs are shown as solid lines and their respective coordination numbers (N(**r**)) are shown as dashed lines with the same colors.


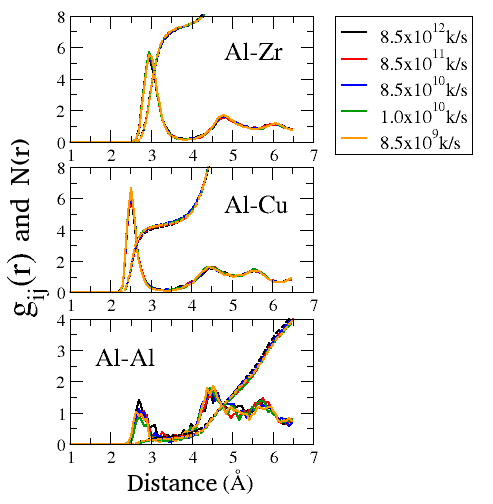


**Supplementary Figure S3**: Relative populations of Al-centered clusters (Al-CCs) with different coordination numbers computed from Voronoi analyses for the BMG Zr_47_Cu_46_Al_7_ prepared via CMD simulations with different cooling rates.


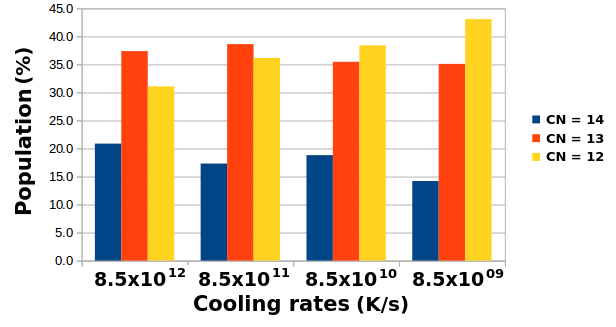


**Supplementary Figure S4**: The time evolution of the absolute populations of Al-CCs with different coordination numbers computed from a Voronoi analysis for the BMG Zr_47_Cu_46_Al_7_ prepared via CMD simulations with different cooling rates. The time interval shown consist of the first 0.2 ps just after the systems reach the room temperature.


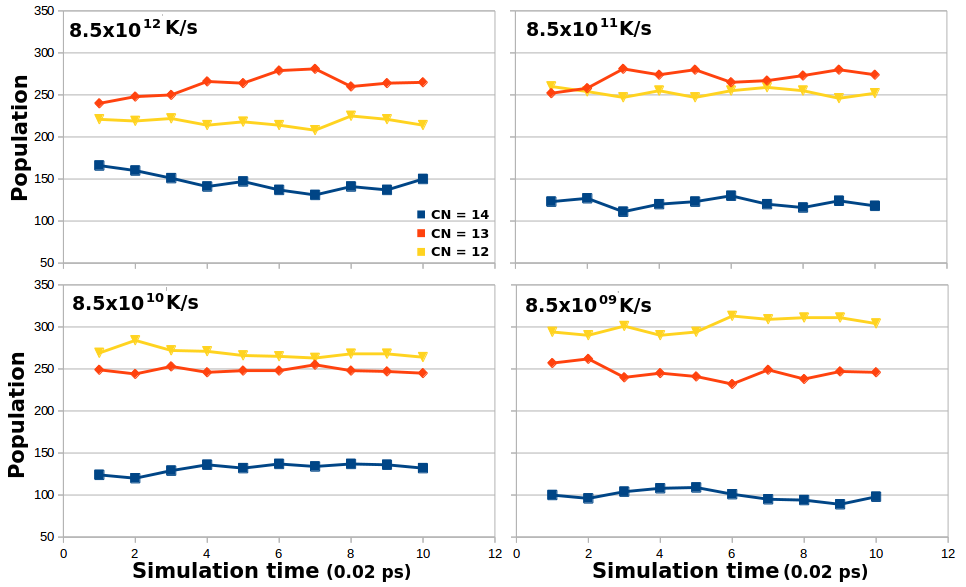


**Supplementary Figure S5**: The cooling curves for the series of BMGs Zr_(50-0.5x)_Cu_(50-0.5x)_Al_x_ (x = 2, 4, 6, 8, 10, 12, and 14) prepared via CMD simulations with a same cooling rate. Each one of the five glass transition temperatures (*T_g_*) in the box at the bottom right corner were computed from the intersection of two fitted lines. The first set of *T_g_*’s were computed as in **Figure S1**. For the second set of *T_g_*’s (marked with an asterisk), each range of points above and below each *T_g_* were chosen automatically in such a way that the respective mean square errors were smaller than 0.0003.

**
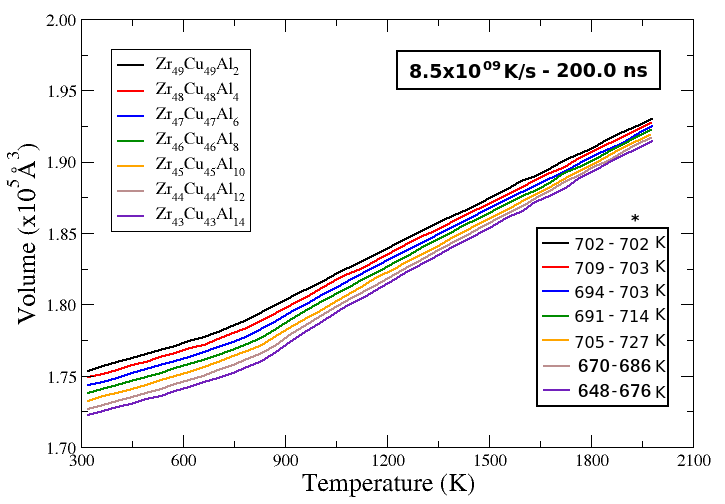
**

**Supplementary Figure S6**: The relative populations of Al-CCs with different first coordination sphere composition index defined as <*n_Zr_*, *n_Cu_*, *n_Al_*>, with *n_S_* the number of first-neighbor atoms (according to the Common Neighbor Analysis) with symbol *S*. These values were computed for the series of BMGs Zr_(50-0.5x)_Cu_(50-0.5x)_Al_x_ (x = 2, 4, 6, 8, and 10) prepared via CMD simulations with a same cooling rate of 8.5×10^9^ K/s.


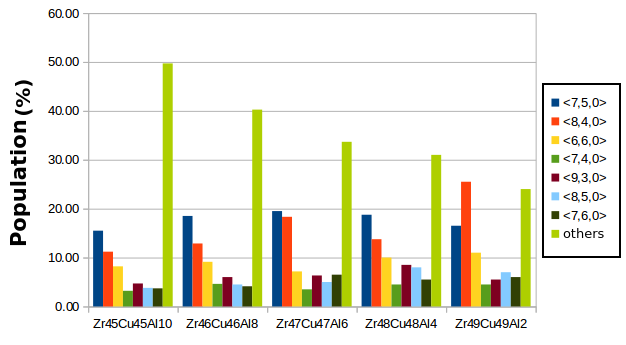


**Supplementary Figure S7**: The relative populations of Al-CCs with different first coordination sphere composition indexes (FCSCIs). These values were computed for the series of BMGs Zr_(50-0.5x)_Cu_(50-0.5x)_Al_x_ (x = 2, 4, 6, 8, and 10) prepared via CMD simulations with a same cooling rate of 8.5×10^9^ K/s. The populations are relative to the total number of Al-CCs with non-zero last number of the FCSCIs, i.e., *n_Al_* > 0.


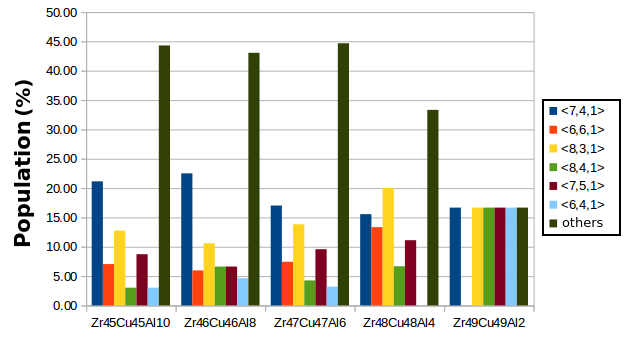


**Supplementary Figure S8**: The relative populations of interconnection regions between two linked Al-CCs, described with an index similar to the first coordination sphere composition index (see **Figure S6**) pointing to the number and types of shared atoms. These values were computed for the series of BMGs Zr_(50-0.5x)_Cu_(50-0.5x)_Al_x_ (x = 2, 4, 6, 8, and 10) prepared via CMD simulations with a same cooling rate of 8.5×10^9^ K/s. An example of three linked Al-CCs is shown.


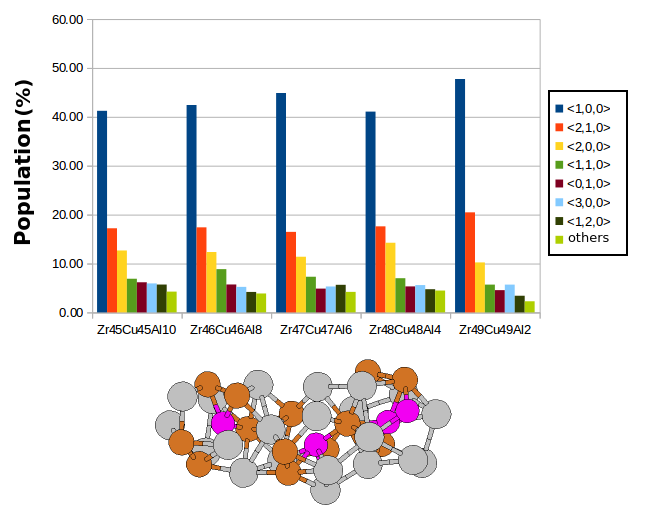


**Supplementary Figure S9**: The relative populations of interconnection regions between two linked Al-CCs, with different number of shared atoms. These values were computed for the series of BMGs Zr_(50-0.5x)_Cu_(50-0.5x)_Al_x_ (x = 2, 4, 6, 8, and 10) prepared via CMD simulations with a same cooling rate of 8.5×10^9^ K/s. An example of three linked Al-CCs is shown.


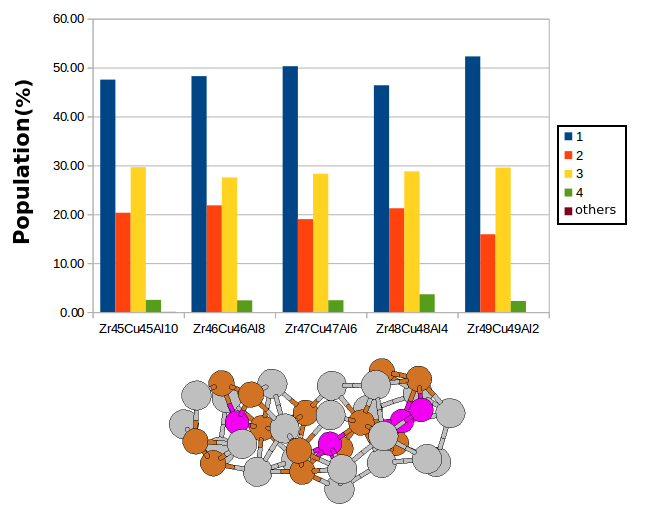


**Supplementary Figure S10**: Dependence of the Fermi contact contribution to the induced magnetic field (**B**_Fc_) on the external magnetic field (**B**_ext_) for the ZrCu_2_Al *Heusler* compound.


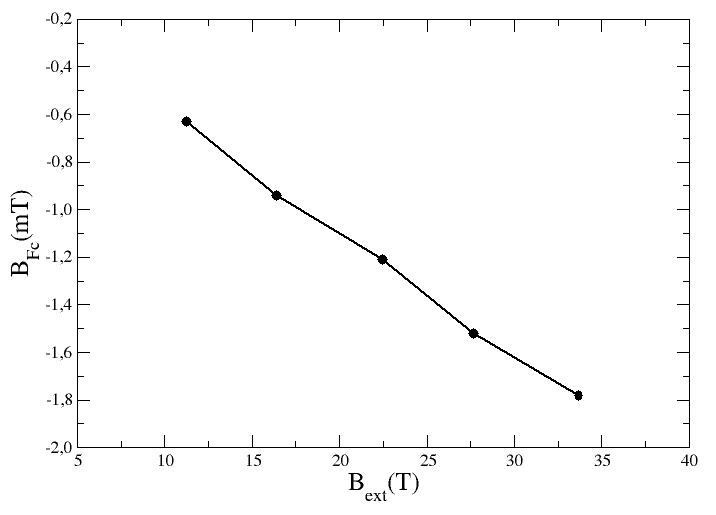


**Supplementary Figure S11**: Dependence of the isotropic orbital (σ_o_) and spin (σ_s_) components of the total shielding on isotropic variations of the volume of the ZrCu_2_Al primitive cell.


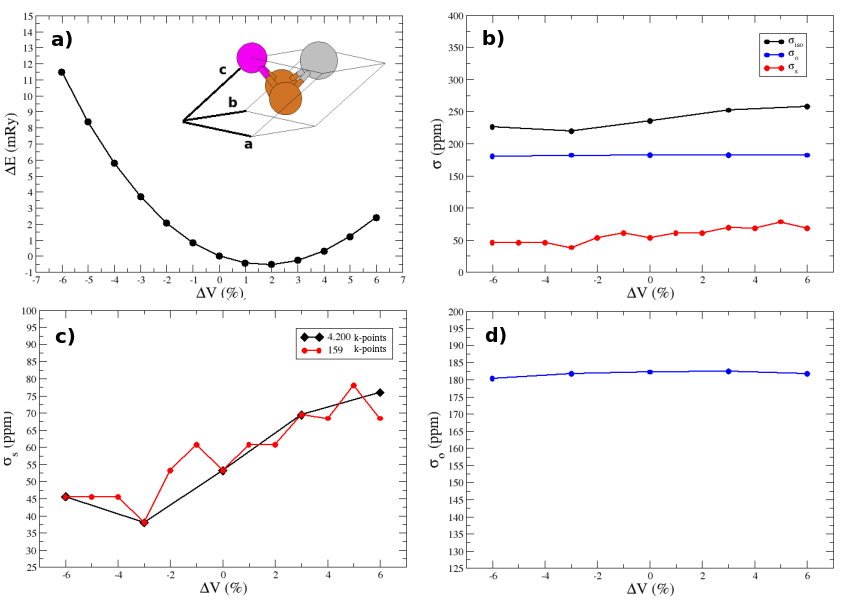


**Supplementary Figure S12**: Set of abstract systems generated from the B2-CuZr structure used in the present work.


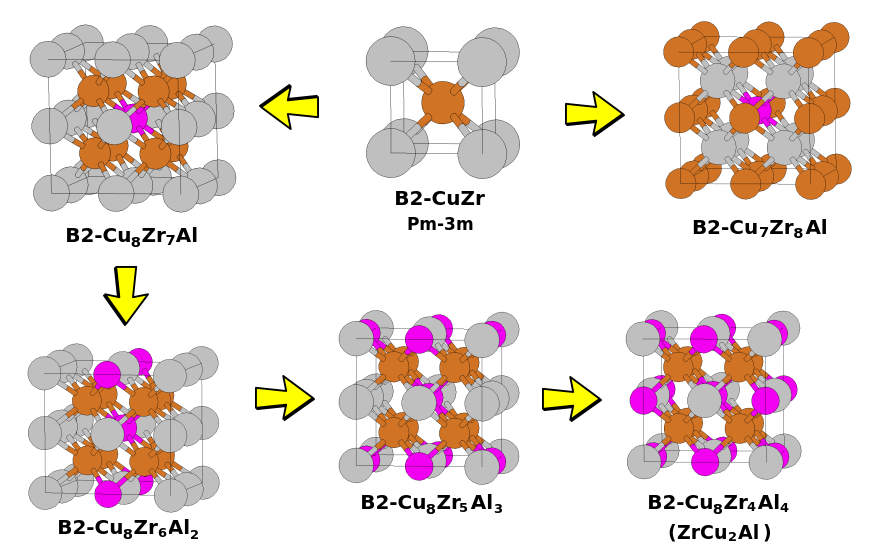


**Supplementary Figure S13**: Projected density of states (PDOS) computed for the abstract system (AS) B2-Cu_8_Zr_6_Al_2_.


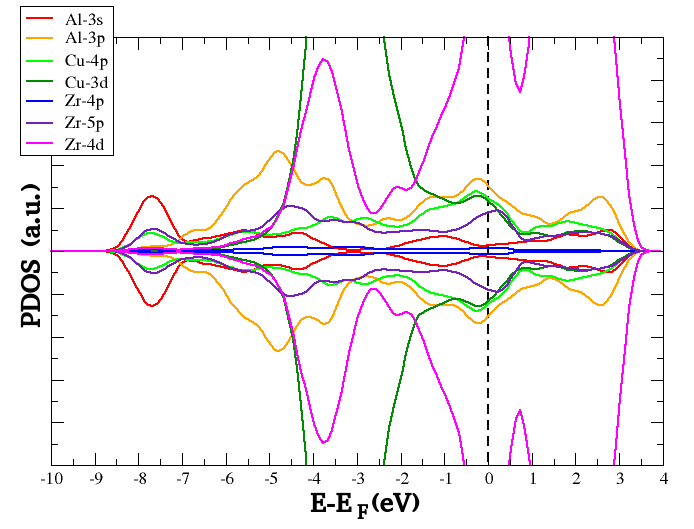


**Supplementary Figure S14**: PDOS computed for the AS B2-Cu_8_Zr_5_Al_3_.


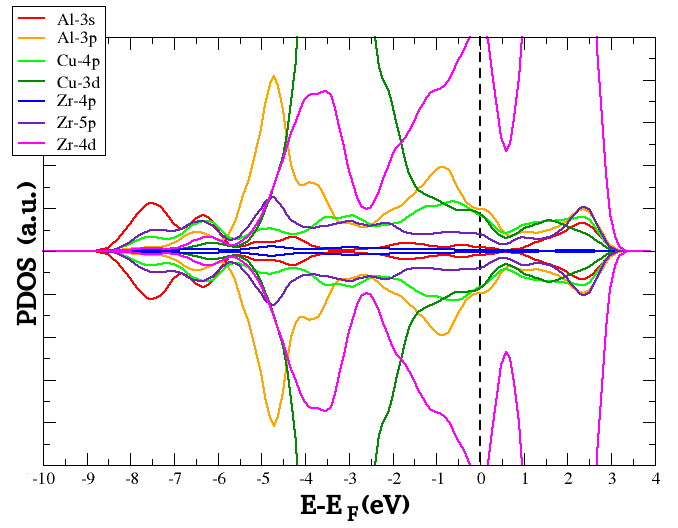


**Supplementary Table S1**: Glass transition temperatures (*T_g_*) and equilibrium mass densities (*ρ_m_*, in grams per cubic centimeter) computed for the system Zr_47_Cu_46_Al_7_. As liquids in thermal equilibrium at 2000 K, and for the respective BMGs at 300 K prepared with different cooling rates (CRs).

| CR (K/s) | *T_g_* (K)*^a^* | *ρ_m_^2000K^* (g/cc) | *ρ_m_^300K^* (g/cc) |
| --- | --- | --- | --- |
| 8.5×10^12^ | 868 | 6.36 | 7.02 |
| 8.5×10^11^ | 808 | 6.36 | 7.03 |
| 8.5×10^10^ | 747 | 6.36 | 7.04 |
| 8.5×10^9^ | 683 | 6.36 | 7.04 |

^a^ From linear fitting as described in Fig. S1.

**Supplementary Table S2**: Glass transition temperatures (*T_g_*) and equilibrium mass densities (*ρ_m_*, in grams per cubic centimeter) computed for the series Zr_(50-0.5_*_x_*)Cu_(50-0.5_*_x_*)Al*_x_*. As liquids in thermal equilibrium at 2000 K, and for the respective BMGs at 300 K prepared with a cooling rate (CR) of 8.5×10^9^ K/s.

| Nominal Composition | *T_g_* (K)*^a^* | *T^*^_g_* (K)*^a^* | *ρ_m_^2000K^* (g/cc) | *ρ_m_^300K^* (g/cc) |
| --- | --- | --- | --- | --- |
| Zr_49_Cu_49_Al_2_ | 702 | 702 | 6.56 | 7.24 |
| Zr_48_Cu_48_Al_4_ | 709 | 703 | 6.49 | 7.16 |
| Zr_47_Cu_47_Al_6_ | 694 | 703 | 6.39 | 7.09 |
| Zr_46_Cu_46_Al_8_ | 691 | 714 | 6.35 | 7.02 |
| Zr_45_Cu_45_Al_10_ | 705 | 727 | 6.26 | 6.94 |
| Zr_44_Cu_44_Al_12_ | 670 | 686 | 6.20 | 6.86 |
| Zr_43_Cu_43_Al_14_ | 648 | 676 | 6.09 | 6.79 |

^a^ From two different linear fitting procedures as described in Fig. S5.

**Supplementary Table S3**: The relative populations of Al-CCs with different coordination numbers and Voronoi indexes.

| CN = 13 | | CN = 14 | |
| --- | --- | --- | --- |
| IV | Population (%) | IV | Population (%) |
| <0,3,6,4,0> | 15,04 | <1,1,8,3,1,0> | 13,27 |
| <0,1,10,2,0> | 36,18 | <2,0,8,2,2,0> | 6,12 |
| <1,2,5,5,0> | 4,47 | <0,3,6,5,0,0> | 11,25 |
| <1,0,9,3,0> | 15,45 | <1,4,3,4,2,0> | 2,04 |
| <0,2,8,3,0> | 5,28 | <1,3,4,5,1,0> | 10,21 |
| <0,4,4,5,0> | 3,25 | <1,3,5,3,2,0> | 4,08 |
| <1,2,6,3,1> | 7,38 | <0,1,10,3,0,0> | 4,08 |
| <1,3,4,4,1> | 2,03 | <2,1,7,2,1,1> | 2,04 |
| <2,2,3,5,1> | 2,03 | <1,2,6,4,1,0> | 6,12 |
|  |  | <0,2,8,4,0,0> | 8,16 |
|  |  | <2,2,4,4,2,0> | 4,08 |
|  |  | <1,1,7,5,0,0> | 5,10 |
|  |  | <1,2,7,2,2,0> | 3,06 |
|  |  | <0,4,4,6,0,0> | 5,10 |
|  |  | <0,4,5,4,1,0> | 2,04 |
